# An Anti‐Fracture and Super Deformable Soft Hydrogel Network Insensitive to Extremely Harsh Environments

**DOI:** 10.1002/advs.202302342

**Published:** 2023-06-08

**Authors:** Baibin Yang, Caihong Wang, Ruihan Xiang, Qiang Zhao, Yong Wu, Shuai Tan

**Affiliations:** ^1^ School of Chemical Engineering Sichuan University No. 24 South Section 1, Yihuan Road Chengdu 610065 China

**Keywords:** anti‐fracture, harsh environments insensitivity, hydrophobic homogenous cross‐linking, soft poly (sodium acrylate) hydrogels, super deformability

## Abstract

Design of hydrogels with superior flexible deformability, anti‐fracture toughness, and reliable environment adaption is fundamentally and practically important for diverse hydrogel‐based flexible devices. However, these features can hardly be compatible even in elaborately designed hydrogels. Herein soft hydrogel networks with superior anti‐fracture and deformability are proposed, which show good adaption to extremely harsh saline or alkaline environments. The hydrogel network is one‐step constructed via hydrophobic homogenous cross‐linking of poly (sodium acrylate), which is expected to provide hydrophobic associations and homogeneous cross‐linking for energy dissipation. The obtained hydrogels are quite soft and deformable (tensile modulus: ≈20 kPa, stretchability: 3700%), but show excellent anti‐fracture toughness (10.6 kJ m^−2^). The energy dissipation mechanism can be further intensified under saline or alkaline environments. The mechanical performance of the hydrophobic cross‐linking topology is inspired rather than weakened by extremely saline or alkaline environments (stretchability: 3900% and 5100%, toughness: 16.1 and 17.1 kJ m^−2^ under saturated NaCl and 6 mol L^−1^ NaOH environments, respectively). The hydrogel network also shows good performance in reversible deformations, ion conductivity, sensing strain, monitoring human motions, and freezing resistance under high‐saline environments. The hydrogel network show unique mechanical performance and robust environment adaption, which is quite promising for diverse applications.

## Graphical abstract

Hydrogels with unique features in soft stretchability and superior anti‐fracture are simply constructed via hydrophobic homogenous cross‐linking. The dynamic hydrophobic dissociations and gradual alignment of polymer chains work collaboratively to provide effective energy dissipation for the hydrogels. The obtained hydrogels are quite soft and deformable, but show excellent anti‐fracture toughness even under extremely harsh saline or alkaline environments.

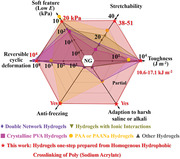

## 1 Introduction

Hydrogels, soft materials with 3D polymer networks confining amounts of water, have received much interest due to their great potential in versatile applications,^[^ ^1^ ^]^ especially for flexible energy devices,^[^ ^1k,l,m^ ^]^ wearable electronics, and ionotronics.^[^ ^1c,d,e,n^ ^]^ These applications require the hydrogels to possess a set of specific properties. For example, hydrogels with low Young's modulus (*E*), good deformability, and high anti‐fracture toughness (*G*) are desirable for the broad applications of flexible electronics to meet the demands of flexibility and durability.^[^ ^1e^ ^]^ However, conventional chemically‐cross‐linked hydrogels can hardly satisfy the customized demands of various applications. Hydrogels resulted from pure physical interactions are usually soft and deformable, but these hydrogels may suffer from poor fracture resistance during applications.^[^ ^2^ ^]^ The anti‐fracture toughness of hydrogels could be significantly improved by introducing various elaborate topologies,^[^ ^3^ ^]^ such as double networks,^[^ ^4^ ^]^ organic‐inorganic nanocomposites,^[^ ^5^ ^]^ slide‐ring connections,^[^ ^6^ ^]^ and crystalline polymeric skeletons,^[^ ^7^ ^]^ into the hydrogels. However, the Young's modulus and strength of hydrogels would be greatly enhanced correspondingly, which sacrificed the soft and deformable features unfavorable for flexible applications. The design of hydrogels with superior flexible deformability and anti‐fracture toughness is of both fundamental and practical importance for diverse hydrogel‐based flexible devices.^[^ ^1e^ ^]^ However, the features of soft compliance and super‐high toughness can hardly be compatible in conventionally designed hydrogels.

Besides the intrinsic excellent mechanical performance, the hydrogels are also required to possess good adaptation under extremely harsh environments for some flexible electronics. For example, hydrogels for flexible ionotronics and electronics should maintain excellent flexibility under high ion concertation to accurately respond to stimulation and transmit signals.^[^ ^1^, ^8^ ^]^ Hydrogels designed for flexible solid‐state metal‐air batteries should retain mechanical stability under strong alkaline electrolytes.^[^ ^9^ ^]^ However, the original mechanical performance of the hydrogels may suffer deterioration in these extremely harsh environments. For example, previous studies have shown that high‐saline environments would induce polymer chain entanglement to enhance the mechanical toughness of the hydrogels.^[^ ^10^ ^]^ But, the stretchability and flexibility of the hydrogel electrolytes would be obviously decreased as the hydrogel networks were hardened under the saline environments. In some situations, tough physical hydrogels based on strong ionic interactions would be ruptured by the high‐saline environments.^[^ ^11^ ^]^ Recently, we showed the great potential of hydrophobic homogenous cross‐linking to fabricate unbreakable poly(acrylamide) (PAAM) hydrogel networks for anti‐fracture and super‐stretchable hydrogels.^[^ ^12^ ^]^ However, the conventional PAAM chains are prone to failure in high‐concentrated alkaline environments for practical applications. Nowadays, hydrogels combining comprehensive properties, including soft flexibility, anti‐fracture toughness, super deformability, and insensitivity to harsh environments, remain to be explored for versatile flexible electronics. We envisioned that a rational design of the polymer network may develop hydrogels satisfying these customized and critical demands, which could also deepen the understanding of hydrogel topology resulted from the hydrophobic homogenous cross‐linking.

**Figure 1:**
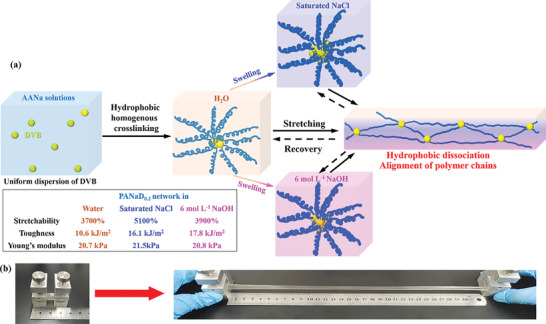
a) Schematic illustration of the energy dissipation mechanism in the hydrogel networks with harsh environments insensitivity, b) ultra‐high stretching images of the prepared hydrogels.

Herein we proposed an anti‐fracture and super deformable hydrogel network with unique mechanical performance, which also showed good adaption to strong saline or alkaline environments. The hydrogel network was simply one‐step constructed via in situ polymerization of aqueous divinyl benzene (DVB) / sodium acrylate (AANa) solutions. The DVB cross‐linkers were well dispersed in the solutions by ultrasonic oscillation, which were supposed to provide hydrophobic associations and homogeneous cross‐linking in the hydrogel network for effective energy dissipation, as shown in **Figure** **1**. The obtained hydrogels were quite soft and deformable (*E*: 20 kPa, stretchability (*ε*_b_): 3700%, Video S1, Supporting Information), but showed excellent anti‐fracture toughness (fracture tensile work (*W*）: 2.3 MJ m^−3^, *G*: 10.6 kJ m^−2^). To the best of our knowledge, similar hydrogels, integrating the soft compliance, ultra‐large deformation, and robust toughness, have rarely been achieved previously. The energy dissipation mechanism would be further enhanced under saline or alkaline environments. Thus, the mechanical performance of the hydrophobic cross‐linking topology was much inspired, rather than weakened, by the extremely saline or alkaline environments (*ε*_b_: 5100%, *G*: 16.1 kJ m^−2^ under saturated NaCl environment; and *ε*_b_: 3900%, *G*: 17.1 kJ m^−2^ under 6 mol L^−1^ NaOH environments, Video S1, Supporting Information), while the soft compliance feature retained (*E*: ≈20 kPa). The hydrogel network also showed good performance in reversible deformations (10^4^ th), ion conductivity (20.85 mS cm^−1^), sensing strain, and freezing resistance under the extremely harsh environments. The hydrogel network was simply prepared from hydrophobic homogeneous cross‐linking of ionic polymers without complicated design, but showed unique outstanding mechanical performance and reliable harsh environment adaption. The work here demonstrated a promising approach to fabricate desirable hydrogels tailored for diverse applications including wearable electronic devices, flexible, and scalable electronic devices.

## 2 Results and Discussion

### 2.1 Hydrogel Preparation

Previously, we showed the great potential of hydrophobic homogenous cross‐linking to fabricate anti‐fracture and super‐stretchable PAAM hydrogel.^[^ ^12^ ^]^ The hydrophobic cross‐linkers were introduced into the acrylamide solutions with the assistance of sodium dodecyl sulfonate (SDS). However, the formation of SDS micelles in solutions was easily disturbed by the factors, such as PH value, temperature, and salinity, leading to an uncontrollable polymerization process. Herein, the hydrogel PANaD_n_ was directly prepared via in situ polymerization of AANa (4.5 mol L^−1^) and DVB (n mol L^−1^, *n* = 0.1, 0.2, 0.3) aqueous solutions without the presence of the SDS micelles. The hydrophobic cross‐linker DVB was conveniently homogenous‐dispersed in the solutions via ultrasonic oscillation of the solutions for 1 h. Before ultrasonic oscillation, the mixture of DVB and AANa solutions was obvious phase separation, as shown in Figure S1a (Supporting Information). The mixture became translucent during ultrasonic oscillation (Figure S1b, Supporting Information). After removing the ultrasonic oscillation, the mixture could still maintain the translucence state for more than 6 h (Figure S1c, Supporting Information). Dynamic light scattering (DLS) measurements revealed that the DVB formed nanodroplets with an average diameter of ≈100 nm in AANa solutions (Figure S2a, Supporting Information). The diameter of DVB nanodroplets was increased with the increase of DVB content at *n* < 0.3, which suggested that DVB cross‐linkers were well dispersed in the solutions. However, the diameter of DVB nanodroplets was almost constant by further adding DVB into solutions, which suggested that the further added DVB was separated from solutions. The maximum content of DVB introduced by ultrasonic oscillation was 0.3 mol L^−1^.

The solution was injected into polytetrafluoroethylene molds with specific shapes, and the polymerization was initiated by ammonium persulphate at 60 °C. After 4 h, transparent hydrogels PANaD_n_ with ≈70 wt.% water were obtained (Figure 1b). The water contents in PANaD_n_ hydrogels were confirmed by a gravimetric method, as described in the Supporting Information. The water contents determined by gravimetric methods for PANaD_0.1_, PANaD_0.2_, PANaD_0.3_ hydrogels were 68.9%, 68.5%, and 67.9%, respectively, which were quite close to the pristine water contents (69.4% for PANaD_0.1_, 69.0% for PANaD_0.2_, 68.4% for PANaD_0.3_ hydrogels).The as‐prepared PANaD_n_ hydrogels showed good water retention due to the moisture absorptivity of the poly (AANa) chains, which only lost less than 10% water after being stored at 80 °C for 1 h (Figure S9a, Supporting Information). DLS measurements (Figure S2b, Supporting Information) of the as‐prepared hydrogels suggested that the DVB nanodroplets disappeared after polymerization. Two new peaks at 2.7 and 960 nm were detected during the DLS measurements of the as‐prepared hydrogels. The cross‐linking nodes based on DVB nanodroplets would further form aggregates with an average size of 960 nm after polymerization due to the hydrophobic interactions, which would provide dynamic reversible physical associations.^[^ ^12^, ^13^ ^]^ Atomic force microscope analysis further confirmed the homogenous aggregations of the DVB nodes in PANaD_n_ hydrogels, as shown in Figure S3 (Supporting Information). Considering that only AANa and DVB were applied during the hydrogel preparation, the peaks at 2.7 nm should be ascribed to the entanglements of the poly (AANa) chains in PANaD_n_ hydrogels due to the ionic interactions. The physical associations and polymer entanglements were supposed to provide effective energy dissipation during the deformation of the hydrogels. Fourier transform infrared spectroscopy (FT‐IR) of freeze‐dried PANaD_n_ further confirmed the successful copolymerization (Figure S4, Supporting Information).^[^ ^14^ ^]^ Considering that only DVB, AANa, and the initiator were contained in the precursor solutions for the PANaD_n_ hydrogel preparation, the fabrication methods here was more convenient and controllable comparing to the previous methods.^[^ ^12^ ^]^

The obtained PANaD_n_ hydrogels showed excellent adhesive performance on diverse surfaces including metals, latex, glass, skin, rock, leaf, and paper, regardless of humidity (Figure S5, Supporting Information), which is quite suitable for wearable, flexible and scalable electronic devices. The strong adhesion of the hydrogels was attributed to the presence of hydrophobic cross‐linking clusters in the PANaD_n_ network.^[^ ^15^ ^]^

Viscoelastic properties of PANaD_n_ hydrogels were characterized by dynamic rheological analysis (Figure S6). The elastic modulus value (*G′*) were much higher than the viscous modulus value (*G′′*), and the solid‐like behavior of PANaD_n_ confirmed the formation of cross‐linked networks in the hydrogels (Figure S6b, Supporting Information). The *G′* increased with the increase in DVB contents, which should be ascribed to the enhanced cross‐linking densities. Loss factor tan *δ* (*G′′*/*G′*) of the PANaD_n_ hydrogels ranged from 0.14–0.45 over the entire measured frequency (Figure S6c, Supporting Information), which were comparable to the tan *δ* (0.17–0.42) measured for PAAM/DVB hydrogels with hydrophobic associations.^[^ ^16^ ^]^ The constructed master curves of tan *δ* increased with the decrease in frequency, and reached a top at frequencies 0.1–0.3 s^−1^, corresponding to a relaxation time of 3.3–10 s.^[^ ^4b^ ^]^ The relaxation time decreased with the increase in DVB contents, which revealed that the hydrophobic associations in the hydrogels were enhanced at a higher DVB contents.

### 2.2 Mechanical Performance of the Hydrogels

**Figure 2:**
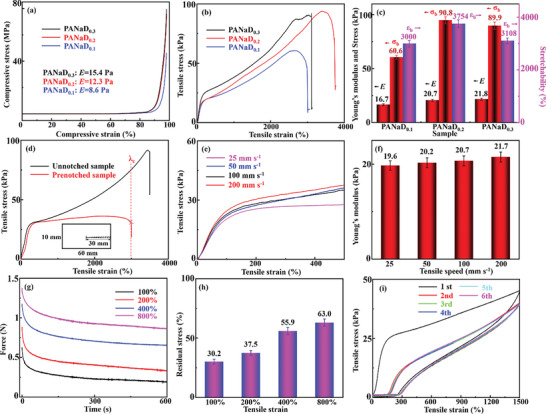
a) Compressive stress‐strain curves of the PANaD_n_ hydrogels; b) Nominal tensile stress‐strain curves of the PANaD_n_ hydrogels; c) *E*, *ε*_b_, and *σ*_b_ as a function of DVB contents for the PANaD_n_ hydrogels; d) Nominal tensile stress‐strain curves of the pre‐notched and unnotched PANaD_0.2_ hydrogels; e) Nominal tensile stress‐strain curves of the PANaD_0.2_ hydrogels at different tensile speeds; f) Young's modulus of PANaD_0.2_ hydrogels at different tensile speeds; g) Stress‐relaxation curves of PANaD_0.2_ hydrogels subjected to different stretch values; h) Residual stresses of PANaD_0.2_ hydrogels after the stress‐relaxation tests; i) Cyclic tensile stress‐strain curves of the PANaD_0.2_ hydrogels at a 1500% strain with no recovery time.

The mechanical performance of hydrogels was measured by a universal test machine. Detailed information about the mechanical performance measurements was described in Supporting Information. Cylinder‐shaped specimens were prepared to perform the uniaxial compression tests. The nominal compressive stress‐strain curves of the PANaD_n_ hydrogels are shown in **Figure** **2**a. The compressive moduli of PANaD_n_ were calculated from the linear region of the stress‐strain curves, as listed in Figure 2a. The PANaD_n_ hydrogels were quite soft at the initial deformations, as the compressive moduli were much less than 100 Pa. However, the hydrogel networks in PANaD_n_ were mechanically robust to withstand compressive stresses, which did not break at a nominal compressive strain of 98%. Both the compressive stresses of PANaD_0.2_ and PANaD_0.3_ at a 98% strain surpassed 70 MPa, which were much higher than the compressive fracture stress of articular cartilage.^[^ ^17^ ^]^ The hydrogel network also showed good elasticity, which could recover to the initial shape upon unloading.

Dumb‐bell‐shaped specimens of PANaD_n_ hydrogels were prepared for the tension tests. The nominal tensile stress‐strain curves of the PANaD_n_ hydrogels are shown in Figure 2b. Tensile behavior of PANaD_n_ hydrogels with different fractions of DVB were similar. The obtained *E*, *ε*_b_, and fracture stress (*σ*_b_) as a function of DVB contents are listed in Figure 2c. All the PANaD_n_ hydrogels were quite soft but super stretchable, which could be stretched up to 37.4 times with a low *E* value of ≈20 kPa. The *E* value of the obtained PANaD_n_ hydrogels were increased with the increase in DVB content, which suggested that the hydrogel network in PANaD_n_ hydrogels became denser at a higher DVB content. PANaD_0.2_ showed balanced Young's modulus, stretchability, and fracture stress among all the prepared PANaD_n_ hydrogels. The *W* during the tension process of PANaD_n_ hydrogels were calculated by integrating the area under the stress‐strain curves. Owing to the superior stretchability, the W value of PANaD_0.2_ hydrogels approached to 2.3 MJ m^−3^, which located in the “super‐tough” regime.^[^ ^18^ ^]^

The anti‐fracture nature and *G* of PANaD_0.2_ hydrogels were further characterized by the pure shear tests and the tear tests using pre‐notched hydrogel samples according to the literature.^[^ ^19^ ^]^ Although PANaD_0.2_ hydrogels were quite soft and weak in Young's modulus, PANaD_0.2_ hydrogels showed super‐high resistance in anti‐crack propagation. The pre‐notched samples (60 mm × 10 mm × 1.5 mm (thickness) with a 30 mm pre‐notch) could still be stretched up to 30 times its original length (Figure 2d), which approached to the unnotched samples. The calculated *G* for the PANaD_0.2_ hydrogels was ≈10.6 kJ m^−2^, which was comparable to the hydrogels possessing extreme mechanical properties^[^ ^3^ ^]^ and orders of magnitude higher than the reported poly (AANa) and poly (acrylic acid) (AA) hydrogels (Table S1, Supporting Information). To the best of our knowledge, PANaD_n_ hydrogels showed the champion stretchability and anti‐fracture toughness among the reported poly (AA) and poly (AANa) hydrogels. The toughness of PANaD_0.2_ hydrogels was also characterized by the tear tests. The obtained tear force‐displacement curves at various tear speeds are shown in Figure S7a (Supporting Information). The tear force increased with the increase in displacement, then reached a plateau. The energy release rates at different tensile speeds were determined using the following equation (*G* = 2*F*/*h*), in which *F* and *h* represent the plateau tensile force and sample thickness. The energy release rates determined from the tear tests at various tear speeds are shown in Figure S7b (Supporting Information). The energy release rate at higher tear speeds approaches to the toughness, while the energy release rate at lower tear speeds is close to the fatigue threshold.^[^ ^19^ ^]^ Thus, the toughness and fatigue threshold of PANaD_0.2_ hydrogels from the tear tests were ≈9.40 and 2.12 kJ m^−2^, respectively.

Based on these results, it could be concluded that PANaD_n_ hydrogels were soft and deformable, but showed superior toughness in anti‐fracture during the compression and tension tests. Typically, hydrogels based on physical interactions, such as hydrophobic associations, are quite soft and deformable, but poor in fracture resistance for practical applications.^[^ ^2^ ^]^ The anti‐fracture toughness of the hydrogels could be improved by introducing versatile elaborate topologies.^[^ ^3^ ^]^ However, the obtained hydrogels would be hardened and the stretchability would obviously deteriorate, which was unfavorable for flexible applications. Nowadays, hydrogels with soft stretchable and anti‐fracture features similar to PANaD_n_ hydrogels have rarely been reported. The PANaD_n_ hydrogels were simply one‐step constructed from solutions of DVB and sodium acrylate without additional complicated design, but showed unique mechanical performance with an integration of soft compliance, superhigh toughness and flexible deformability in one hydrogel, which paved a new way to fabricate hydrogels combining soft and tough features.

### 2.3 Energy Dissipation Mechanism of the Hydrogels

The PANaD_n_ hydrogels showed unique mechanical performance with soft compliance, superhigh toughness, and deformability, which was quite different from the unconventional hydrogel networks with extreme properties.^[^ ^3^ ^]^ Additional tensile tests, X‐Ray diffraction (XRD) measurements, and polarized optical microscope (POM) observations have been performed to further analyze the mechanism of the unique mechanical performance for the hydrogels.

Typically, Young's moduli of hydrogels varied significantly with the tensile speeds when the deformations of the hydrogels were dominated by the dynamic and reversible physical interactions.^[^ ^2^, ^11^ ^]^ In contrast, the Young's moduli of hydrogels cross‐linked by covalent bonds were independent of the strain rates because the strain rates were slower than the deformation rates of the polymer network.^[^ ^20^ ^]^ Thus, tension tests at various displacement rates were performed for PANaD_0.2_ hydrogels to investigate the energy dissipation mechanism in the hydrogels (Figure 2e). The Young's moduli obtained from tensile stress‐strain curves at different tensile deformation rates are shown in Figure 2f. The *E* value of PANaD_0.2_ hydrogels increased slightly from 19.6 ± 0.9 to 21.7 ± 1.0 kPa with the increase in tensile rates from 25 to 200 mm s^−1^, which was quite different from the hydrogels obtained by pure physical cross‐linking or chemical cross‐linking. This result implied that the deformation of the PANaD_0.2_ hydrogels was contributed by both dynamic physical cross‐linking of physical interactions and covalently chemical cross‐linking of poly (AANa) chains. Considering that the *E* value of PANaD_0.2_ hydrogels only slightly increased with the increase in tensile rates, the influence of physical interactions was supposed to be weaker than the covalent chemical cross‐linking for the PANaD_0.2_ hydrogels.

Stress relaxation tests were also conducted to analyze the deformation process of the hydrogels (Figure 2g). Stress relaxation curves of PANaD_0.2_ hydrogels subjected to 100%, 200%, 400%, and 800% tensile strains are shown in Figure 2g. The residual stresses after 600 s are shown in Figure 2h. PANaD_0.2_ hydrogels subjected to 100% strains retained 30.3% initial stress value, which showed a liquid‐like relaxation behavior. The liquid‐like behavior of PANaD_0.2_ hydrogels at initial deformation should be mainly contributed by physical interactions of hydrophobic associations.^[^ ^19^, ^21^ ^]^ Nevertheless, the residual stresses increased with the increase in tensile strains of PANaD_0.2_ hydrogels. PANaD_0.2_ hydrogels subjected to 800% tensile strains exhibited a solid‐like relaxation behavior, which retained ≈63% stress value after 600 s.^[^ ^21^ ^]^ The enormous differences in residual stresses (Figure 2h) indicated two distinct relaxation mechanisms in PANaD_0.2_ hydrogels. The extension of the covalently cross‐linked polymer network in PANaD_0.2_ hydrogels gradually dominated at a larger deformation, which induced the solid‐like relaxation behavior.

Cyclic tensile tests at room conditions without special treatment were further performed to investigate the recoverability of the PANaD_0.2_ hydrogels. The cyclic tensile stress‐strain curves of PANaD_0.2_ hydrogels at a 1500% tensile strain with no recovery interval are shown in Figure 2i. After the first loading‐unloading process, an obvious hysteresis with ≈20% residual strain was detected in the following cyclic tests. The hysteresis ratio of the PANaD_0.2_ hydrogels was much lower than that of the physically cross‐linked hydrogels.^[^ ^11^ ^]^ The hysteresis should be ascribed to the dissociations of the hydrophobic domains. Despite the hysteresis, the hydrogels were partially recovered immediately with no recovery time, which still retained the reversible elastic behavior in the successive loading‐unloading cycles. The *E* value of the hydrogels during the second loading‐unloading curve was ≈15 kPa, which was slightly smaller than that obtained from the first loading. This result suggested that the recovery of PANaD_0.2_ hydrogels involves both a quick process and a slow process.^[^ ^11^ ^]^ This two‐stage recovery process was probably related to the competition between the elasticity of the polymer chains and the strength of the hydrophobic reassociations during the unloading process. The hydrophobic associations would be ruptured during the first loading, while the polymer chains were only elastically elongated. During the initial recovery, the elastic contraction of the polymer chains in PANaD_0.2_ hydrogels was dominant, and led to the quick recovery. At the following recovery, the elastic contraction became weak and the reassociations of hydrophobic domains slowed down the recovery of the primary networks to the equilibrium state, which induced the hysteresis. The following cyclic stress‐strain curves of the PANaD_0.2_ hydrogels almost coincided with the second cycle, which indicated that the hydrogel had a robust self‐recovery ability. Meanwhile, the PANaD_0.2_ hydrogels only showed slight hysteresis at an applied stretch of *λ* = 2 for over 10^4^ cyclic tensile loads owing to the quick recovery of the polymer network (Video S2, Supporting Information). These results also demonstrated that that the deformation of PANaD_0.2_ hydrogels was contributed by both dynamic physical cross‐linking of hydrophobic interactions and covalently chemical cross‐linking of the poly (AANa) chains.

**Figure 3:**
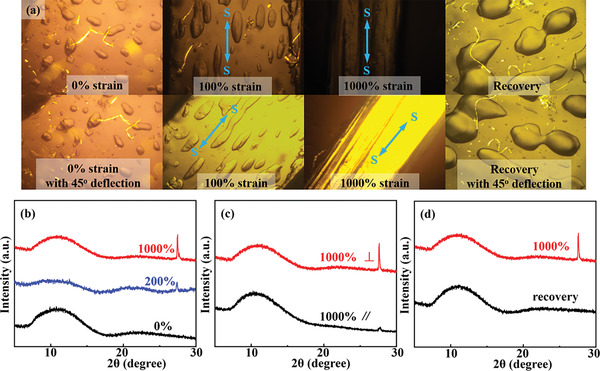
a) Polarized microscopic image of the PANaD_0.2_ hydrogels under various states (up) and with 45^o^ deflection (down), S: stretching; b) XRD patterns of the PANaD_0.2_ hydrogels freeze‐dried at different strain with the incident the beam perpendicular to the stretching direction, c) XRD patterns of the PANaD_0.2_ freeze‐dried at 1000% strain with incident beam perpendicular (⊥) and along (∥) to the stretching direction. d) XRD patterns of the PANaD_0.2_ freeze‐dried at 1000% strain and recovered PANaD_0.2_ after being stretched to 1000% strain (PANaD_0.2_ was stretched to 1000% strain, then freeze‐dried immediately after the load was removed to obtain the recovered sample).

POM observations were also performed to characterize the deformation process of the polymer networks. As shown in **Figure** **3**a, the unstretched PANaD_n_ hydrogels showed no brightness change on rotation of the samples under POM observations, which confirmed that the polymer network in PANaD_0.2_ hydrogels was isotropic. Once being stretched, the hydrogels showed obvious periodic brightness change on each rotation of the samples by 45° under the POM observations (Figure 3a; and Video S3, Supporting Information).^[^ ^22^ ^]^ The brightness contrasts during the rotation were enhanced with the increase in tensile deformations of PANaD_0.2_ hydrogels. This result revealed that the polymer networks in PANaD_n_ hydrogels became anisotropic, which should be ascribed to the gradual unfolding of poly (AANa) chains along the stretching direction. Since the poly (AANa) chains were homogeneously cross‐linked by DVB cross‐linkers, the polymer chains would uniformly extend to full alignment during being stretched, which achieved the superhigh stretchability of the hydrogels. The gradually aligned poly (AANa) chains would reinforce the structural integrity of hydrogels to tolerate the external force for the ultra‐high fracture resistance. Thus, the pre‐notched samples were also super stretchable during the pure shear tests.^[^ ^12^ ^]^ The periodic brightness change of the hydrogels under the POM observations would be disappeared once the tensile force was released (Figure 3a), which revealed that the poly (AANa) chains in the PANaD_0.2_ hydrogels would reversibly transform between the tangled coil state and the aligned state during being stretched and released. The reversible changes of the hydrogel network guaranteed the partially recoverable deformation of the PANaD_n_ hydrogels. Pure poly (AANa) hydrogels in absence of DVB cross‐linker were also prepared with procedure similar to PANaD_n_ hydrogels. The pure poly (AANa) hydrogels showed results similar to the PANaD_n_ hydrogels under the POM observations (Figure S8c, Supporting Information), which further demonstrated that the deformation of the PANaD_n_ hydrogels was contributed by the gradual unfolding of poly (AANa) chains along the stretching direction.

The topology change of hydrogel networks during deformations was further verified by XRD patterns of the PANaD_0.2_ hydrogels freeze‐dried at different deformations (Figure 3b). Only a broad diffraction peak at 2*θ* ≈ 10.0^o^ was observed for the unstretched sample, wherein the polymer chains were under a tangled coil state. The tangled coil state of polymer chains in the PANaD_0.2_ hydrogels was disturbed once the hydrogel network was being stretched to a 50% strain, as a sharp diffraction peak at 2*θ* ≈ 26.0^o^ appeared.^[^ ^7^, ^12^ ^]^ This result revealed that the polymeric skeletons in the PANaD_0.2_ hydrogels were deformed once being stretched (Figure 3b). The peaks were intensified with the increase in stretch values due to the gradual deformation of the polymer networks in hydrogels. XRD measurements with incident beam perpendicular and along the stretch direction were performed for PANaD_0.2_ hydrogels freeze‐dried at *λ* = 10 to further validate the direction of the polymer chains ordering (Figure 3c). The intensity of the diffraction peaks was much weaker when the incident beam was along to the stretching direction. The difference in the XRD patterns was supposed to be the result of anisotropic polymeric skeletons in stretched PANaD_0.2_ hydrogels. The peak representing the ordering change of poly (AANa) chains disappeared for the PANaD_0.2_ samples freeze‐dried immediately after recovery from *λ* = 10 (Figure 3d), which demonstrated that the extended poly (AANa) chains recovered to a lower energy coiled state rapidly like rubber. These results also demonstrated that the polymeric topology in PANaD_0.2_ hydrogels could recover reversibly from the ultra‐large deformations. The gradual deformation of the polymer networks along the stretching direction would effectively dissipate energy to tolerate the external force, which induced a higher toughness of the hydrogels to resist fracture.^[^ ^12^ ^]^ According to the POM and XRD results, the ultra‐large stretchability and superior anti‐fracture of PANaD_n_ hydrogels should be originated from the gradual alignment of the poly (AANa) chains along the elongation direction.

Based on these results above, it could be concluded that the PANaD_n_ chains would be dragged to deform accompanying with the dissociations of hydrophobic cross‐linking nodes during the tensile deformation of PANaD_n_ hydrogels. The PANaD_n_ hydrogels were fabricated via hydrophobic homogenous cross‐linking strategy. The hydrophobic cross‐linkers were supposed to provide hydrophobic associations and homogeneous cross‐linking in the networks of present hydrogels and our previously reported PAAM hydrogels.^[^ ^12^ ^]^ Both the hydrogels were anti‐fracture and super‐stretchable, which highlighted that the hydrophobic homogenous cross‐linking strategy was a general and scientific principle to develop state‐of‐art hydrogels. However, although both the hydrogel networks were fabricated via a similar strategy, it was quite interesting that the energy dissipation mechanisms for both hydrogels during deformations were quite different. The PAAM hydrogels showed two individual deformation process, which worked step‐by‐step to dissipate energy during the deformations. The initial deformation of the PAAM hydrogels was contributed by the dissociations of hydrophobic domains, which did not disturb the tangled coil state of the PAAM chains. After that, the following large deformation of the PAAM hydrogels was sustained by the quick unfolding of the coiled PAAM chains. In contrast, both the unfolding of the poly (AANa) chains and hydrophobic dissociations contributed together, rather than individually, to dissipate energy during the initial deformations of the PANaD_n_ hydrogels. This result demonstrated for the first time that the type of polymers could influence the energy dissipation mechanism in the hydrogels resulted from hydrophobic homogenous cross‐linking. The distinct deformation process implied that the strength of the PANaD_n_ chains was much weaker than that of the PAAM chains, which induced the soft feature of the PANaD_n_ hydrogels. Besides, the ionic polymer chains of poly (AANa) in PANaD_n_ hydrogel networks were supposed to provide additional superior adaption to strong ionic environments.

### 2.4 Hydrogels Insensitive to Harsh Environments

The stability of the PANaD_n_ hydrogel network in extremely harsh environments including strong saline, strong alkaline, and freezing environments was further investigated. The PANaD_0.2_ were first freeze‐dried, then the freeze‐dried hydrogel networks were immersed into saturated NaCl solutions or 6 mol L^−1^ NaOH solutions at room temperature until the hydrogel networks recovered to the original volume. The swelling behavior of freeze‐dried PANaD_0.2_ networks in saturated NaCl solutions or 6 mol L^−1^ NaOH solutions are shown in Figure S9b (Supporting Information). The hydrogels swelling NaCl solutions and 6 mol L^−1^ NaOH solutions were labeled as PANaD_0.2_‐NaCl and PANaD_0.2_‐NaOH, respectively. The PANaD_0.2_‐NaCl and PANaD_0.2_‐NaOH hydrogels were prepared by immersing freeze‐dried PANaD_0.2_ network into saturated NaCl and 6 mol L^−1^ NaOH solutions for ≈3 and 6 h, respectively. Both the hydrogels were transparent. Viscoelastic properties of PANaD_0.2_‐NaCl and PANaD_0.2_‐NaOH hydrogels were also characterized by dynamic rheological analysis (Figure S6, Supporting Information). The *G′* values were much higher than the *G′′* values within the measured range for both hydrogels. Also, the *G′* values were enhanced after introducing NaCl or NaOH into the hydrogels during the rheological analysis. These results indicated that the hydrogel network in PANaD_0.2_ was highly adaptive to the strong saline or strong alkaline environments.

**Figure 4:**
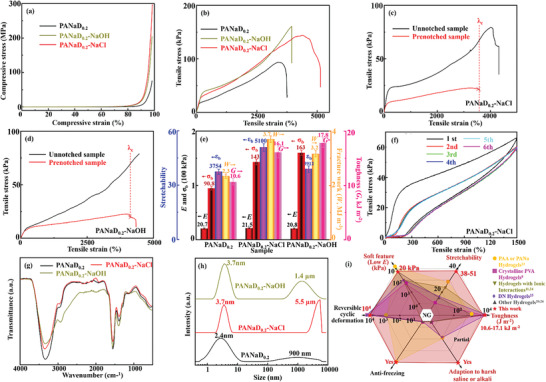
a) Compressive stress‐strain curves of the PANaD_0.2_, PANaD_0.2_‐NaCl, PANaD_0.2_‐NaOH hydrogels; b) Nominal tensile stress‐strain curves of the PANaD_0.2_, PANaD_0.2_‐NaCl, PANaD_0.2_‐NaOH hydrogels; c) Nominal tensile stress‐strain curves of the pre‐notched and unnotched PANaD_0.2_‐NaCl hydrogels; d) Nominal tensile stress‐strain curves of the pre‐notched and unnotched PANaD_0.2_‐NaOH hydrogels; e) *E*, *ε*_b_,*σ*_b_, *W* and *G* of the PANaD_0.2_, PANaD_0.2_‐NaCl, PANaD_0.2_‐NaOH hydrogels; f) Cyclic tensile stress‐strain curves of the PANaD_0.2_‐NaCl hydrogels at a 1500% strain with no recovery time; g) FT‐IR spectra of freeze‐dried PANaD_0.2_, PANaD_0.2_‐NaCl, PANaD_0.2_‐NaOH; h) The size distributions of PANaD_0.2_, PANaD_0.2_‐NaCl, PANaD_0.2_‐NaOH hydrogels; i) Comprehensive performance comparison in stretchability, Young's modulus, fracture toughness, reversible cyclic deformation, and environment adaption between PANaD_0.2_ hydrogels and the representative hydrogels with extreme mechanical properties in recent years. (The data shown in Figure 4f are based on the best paraments for each characteristic from a series of hydrogels, NG: not given).^[^ ^7^, ^11^, ^20^, ^23^, ^24^, ^25^, ^26^ ^]^

The mechanical performance of the PANaD_0.2_ hydrogel network in strong saline and strong alkaline environments was further studied. The nominal compressive stress‐strain curves of the PANaD_0.2_, PANaD_0.2_‐NaCl, and PANaD_0.2_‐NaOH hydrogels are shown in **Figure** **4**a. The compressive stress‐strain curves of PANaD_0.2_‐NaCl and PANaD_0.2_‐NaOH were similar to that of PANaD_0.2_, as the compressive moduli were quite low. Both PANaD_0.2_‐NaCl and PANaD_0.2_‐NaOH could withstand more than 200 MPa compressive stress at a stain of 98% without breakage, which indicated that the hydrogel network was strengthened in these harsh environments to resist compressive stresses.

The nominal tensile stress‐strain curves of the PANaD_0.2_, PANaD_0.2_‐NaCl, and PANaD_0.2_‐NaOH hydrogels are shown in Figure 4b. The anti‐fracture toughness of PANaD_0.2_‐NaCl and PANaD_0.2_‐NaOH hydrogels were also characterized by pure shear tests and the tear tests, as shown in Figure 4c,d, and Figure S7c–e (Supporting Information). The obtained Y*E*, *ε*_b_,*σ*_b_, *W*, and *G* for PANaD_0.2_, PANaD_0.2_‐NaCl, and PANaD_0.2_‐NaOH hydrogels were summarized and are shown in Figure 4e. Both PANaD_0.2_‐NaCl and PANaD_0.2_‐NaOH retained the soft feature with *E* of ≈20 kPa, which were slightly higher than that of PANaD_0.2_. Surprisingly, both the *ε*_b_ and *σ*_b_ of PANaD_0.2_‐NaCl and PANaD_0.2_‐NaOH were much enhanced comparing to those of PANaD_0.2_ (Figure 4b). As a result, the *W* was also promoted. Meanwhile, the strong saline or alkaline environments did not disturb the ability of anti‐crack propagation performance of the PANaD_0.2_ network. The *ε*_b_ value of the pre‐notched samples for PANaD_0.2_‐NaCl and PANaD_0.2_‐NaOH hydrogels was quite close to those of the unnotched samples (Figure 4c,d). The calculated *G* from pure shear tests of PANaD_0.2_‐NaCl and PANaD_0.2_‐NaOH hydrogels was 16.12 and 17.83 kJ m^−2^, respectively, which were much higher than that of PANaD_0.2_ hydrogels (10.60 kJ m^−2^). Similar toughness intensification was also observed for PANaD_0.2_‐NaCl and PANaD_0.2_‐NaOH hydrogels from the tear tests (Figure S7c–e, Supporting Information). The calculated *G* from the tear tests of PANaD_0.2_‐NaCl and PANaD_0.2_‐NaOH hydrogels was 14.44 and 24.04 kJ m^−2^, respectively, which were much higher than that of PANaD_0.2_ hydrogels (9.40 kJ m^−2^). The fatigue threshold of PANaD_0.2_‐NaCl and PANaD_0.2_‐NaOH hydrogels from tear the tests were ≈1.88 and 2.47 kJ m^−2^, respectively.

PANaD_0.2_‐NaCl and PANaD_0.2_‐NaOH hydrogels also showed good anti‐freezing properties, which maintained the excellent tensile performance after being stored at −20 °C for 24 h. (Video S4, Supporting Information). Differential scanning calorimetry (DSC) measurements were conducted to confirm the anti‐freezing properties of the hydrogels (Figure S17, Supporting Information). No obvious exothermic peak was observed for the DSC traces of the PANaD_0.2_‐NaCl hydrogels from 30 to −60 °C, which suggested that the PANaD_0.2_‐NaCl hydrogels was not freezing within the measured temperatures. In contrast, an exothermic peak at ≈−52.6 °C was observed for PANaD_0.2_ hydrogels, which corresponded to the freezing point of PANaD_0.2_ hydrogels. These results demonstrated that the good anti‐freezing performance of PANaD_0.2_‐NaCl and PANaD_0.2_‐NaOH hydrogels was mainly contributed by the high contents of NaCl or NaOH in the hydrogels, which might form a strong hydration effect with free water to improve the anti‐freezing performance of the hydrogels.^[^ ^8b^ ^]^ All these results demonstrated that the mechanical performance of the PANaD_0.2_ network was further inspired rather than weakened by the strong saline or strong alkaline environments. Cyclic tensile stress‐strain curves of PANaD_0.2_‐NaCl and PANaD_0.2_‐NaOH hydrogels were also performed to investigate the recoverability of the hydrogels (Figure 4f; Figure S10, Supporting Information). The result suggested that the PANaD_0.2_‐NaCl and PANaD_0.2_‐NaOH hydrogels also showed recovery processes similar to the PANaD_0.2_ hydrogels, which revealed that deformation of PANaD_0.2_‐NaCl and PANaD_0.2_‐NaOH hydrogels were contributed by both dynamic cross‐linking of physical interactions and covalently chemical cross‐linking of poly (AANa) chains. The mechanical performance of PANaD_0.2_‐NaCl and PANaD_0.2_‐NaOH hydrogels remained after storing the hydrogels under the seal state for 2 months (Figure S11, Supporting Information). This suggested that both PANaD_0.2_‐NaCl and PANaD_0.2_‐NaOH hydrogels showed long‐termed stability in the extremely saline and alkaline environments, which have great potential toward practical applications.

Previous studies have shown that the traditional hydrogel polymer networks, such as PVA, PAAM and chitosan, would be entangled under a NaCl environment during hydrogel preparation due to the salting‐out effect.^[^ ^10^ ^]^ The chain entanglements would provide additional physical cross‐linking to significantly promote the fracture stresses and Young's moduli, but decrease the stretchability of the hydrogels. In contrast, both the stretchability and fracture stress of the PANaD_0.2_ network were much improved under strong saline and alkaline environments, while the Young's moduli were only slightly enhanced. These phenomena implied that the energy dissipation mechanism of the PANaD_0.2_ network under strong saline and alkaline environments was different from that of the reported hydrogels.

FT‐IR measurements, DLS measurements, XRD measurements, and POM observations were further performed to analyze the intensification mechanism of the tensile behavior of PANaD_0.2_ network under strong saline and alkaline environments. The FT‐IR spectra of freeze‐dried PANaD_0.2_, PANaD_0.2_‐NaCl, and PANaD_0.2_‐NaOH were almost identical (Figure 4g), which revealed no new bond and no chemical shift was formed in the hydrogel networks after introducing NaCl or NaOH. This result indicated that the PANaD_0.2_ network was chemically stable in strong saline and alkaline environments, and no additional chemical cross‐linking or physical cross‐linking was formed in PANaD_0.2_‐NaCl and PANaD_0.2_‐NaOH hydrogels. XRD measurements (Figure S12, Supporting Information) and POM observations (Figure S8, Supporting Information) revealed that the poly (AANa) chains in PANaD_0.2_‐NaCl and PANaD_0.2_‐NaOH hydrogels would also be gradually aligned during tensile deformation. Also, the *E* value of PANaD_0.2_‐NaCl and PANaD_0.2_‐NaOH hydrogels increased slightly with the increase in tensile rates (Figure S13, Supporting Information). These results were consistent with that from PANaD_0.2_ hydrogels, which revealed that the energy dissipation mechanisms in PANaD_0.2_‐NaCl and PANaD_0.2_‐NaOH during the deformations remained the same as that in PANaD_0.2_ hydrogels, which was not disturbed by the harsh environments. The homogenous cross‐linking of poly (AANa) chains ensured the anti‐fracture feature of PANaD_0.2_‐NaCl and PANaD_0.2_‐NaOH hydrogels. Despite these, DLS measurements detected that the size distribution of the PANaD_0.2_ network was changed in PANaD_0.2_‐NaCl and PANaD_0.2_‐NaOH hydrogels, as shown in Figure 4h. The average hydraulic diameters of the DVB aggregations were much enhanced in PANaD_0.2_‐NaCl and PANaD_0.2_‐NaOH hydrogels because the hydrophobic associations of the DVB nodes would be increased under strong polar environments. The average hydraulic diameters of the poly (AANa) entanglements in both hydrogels were also slightly enlarged, which implied that more poly (AANa) chains were entangled due to the enhanced ionic interactions under strong polar environments. Meanwhile, the strains at the yield points for PANaD_0.2_‐NaCl and PANaD_0.2_‐NaOH hydrogels were much enhanced comparing to that of PANaD_0.2_ hydrogel (Figure 4b). These results suggested that the PANaD_0.2_ network formed a denser assembly under the strong polar environments comparing to the original assembly of PANaD_0.2_ hydrogels. Thus, the stretchability of PANaD_0.2_‐NaCl and PANaD_0.2_‐NaOH hydrogels was also promoted accordingly. The enhanced hydrophobic interactions and ionic interactions would provide an extra energy dissipation, which advanced the fracture resistance during the deformations. As a result, the mechanical performance of the PANaD_0.2_ network was further inspired in PANaD_0.2_‐NaCl and PANaD_0.2_‐NaOH hydrogels.

Compared to the representative poly (AA), poly(AANa) hydrogels (Table S1, Supporting Information), and other hydrogels (Table S2, Supporting Information) with extreme mechanical properties reported in recent years, PANaD_0.2_ hydrogel networks showed an outstanding comprehensive mechanical performance in softness (low Young's modulus), stretchability, fracture work, toughness, extremes harsh environments insensitivity and reversible cyclic deformations (Figure 4i). The unique outstanding feature of PANaD_n_ hydrogel networks demonstrated a quite promising approach to fabricating desirable hydrogels tailored for diverse applications including wearable electronic devices, and flexible and scalable electronic devices.

### 2.5 Hydrogel Network for Flexible Electronics

**Figure 5:**
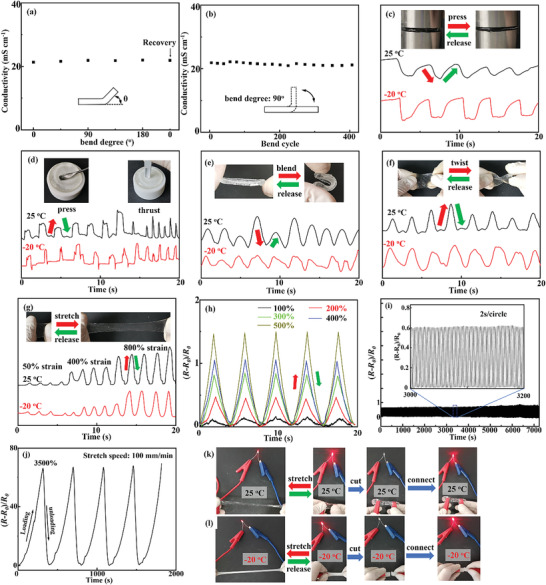
a) Ion conductivity of the PANaD_0.2_‐NaCl hydrogels being bended at various degrees; b) Ion conductivity of the PANaD_0.2_‐NaCl hydrogels during cyclic bending; c–g) resistance change of the PANaD_0.2_‐NaCl hydrogels at room temperature and −20 °C during deformation: c) compress and release, d) press or thrust and release, e) bend and release, f) twist and release, g) stretch and release; h) resistance change of the PANaD_0.2_‐NaCl hydrogels during cyclic tensile deformation at different strains; i) resistance change of the PANaD_0.2_‐NaCl hydrogels during 3600^th^ cyclic deformation (200% strain); j) resistance change of the PANaD_0.2_‐NaCl hydrogels during large cyclic deformation (3500% strain); k,l) bright change of LED controlled by deformation of the PANaD_0.2_‐NaCl hydrogels at room temperature k) and −20 °C l).

The PANaD_0.2_ network is quite suitable for developing hydrogels toward flexible and wearable electronics due to the outstanding features including soft, super deformable, anti‐fracture, and insensitiveness to the harsh environments. PANaD_0.2_‐NaCl hydrogels were applied as strain sensors to verify the potential of the PANaD_0.2_ network for flexible and wearable electronics. The saturated NaCl ions were served for electronic transmission of hydrogel sensors. The PANaD_0.2_‐NaCl hydrogels were sandwiched between two aluminum foils to investigate the conductivity variation under different bending states (**Figure** **5**a,b). The conductivities of the hydrogels under different bending states were determined by electrochemical impedance spectroscopy measurements. Detailed information and the obtained Nyquist plots are shown in the Supporting Information (Figure S14, Supporting Information). The PANaD_0.2_‐NaCl hydrogels showed good conductivity (20.85 mS cm^−1^) independent to the bending angles, and the conductivity was stable after the successive 400 bending cycles at 90^o^. These results indicated that the PANaD_0.2_‐NaCl hydrogels possessed good conducting and anti‐fatigue performance for flexible electronics. Similar performance could also be achieved by PANaD_0.2_‐NaOH hydrogels.

The conductivity changes of PANaD_0.2_‐NaCl hydrogels during deformations could be used to sense the strains. The real‐time resistances of PANaD_0.2_‐NaCl hydrogels accompanying with the deformations were measured, and the strain sensitivity of PANaD_0.2_‐NaCl hydrogels was further evaluated by the relative resistance change ((*R*‐*R*_0_)/*R*_0_) to the applied deformation, where *R*_0_ and *R* are the initial resistance and the resistance at a certain stretch value. The transient of the electrical current for PANaD_0.2_‐NaCl hydrogels during sudden deformation was monitored, and the response and recovery time of PANaD_0.2_‐NaCl sensors are ≈140 ms, which was sufficient accuracy for sensing various strains (Figure S15a, Supporting Information). As shown in Figure 5c–g, the resistances of PANaD_0.2_‐NaCl hydrogels changed obviously when the hydrogels were stretched, compressed, thrusted, bended, and twisted. The resistances recovered reversibly when the stresses were released. These results suggested that PANaD_0.2_‐NaCl hydrogels were robust as strain sensors to detect multiple deformations.

The resistance changes of PANaD_0.2_‐NaCl hydrogels under cyclic stretching‐relaxing at various tensile deformations from 100% to 500% are shown in Figure 5h. The resistance changes of PANaD_0.2_‐NaCl hydrogels were reliable and repeatable during the cyclic deformations and their electrical resistance completely recovered upon releasing of the tensile strain in each cycle. The resistance changes at different tensile strains from 100% to 500% were summarized in Figure S15b (Supporting Information), which varied linearly as a function of strains. The linear change in resistance of PANaD_0.2_‐NaCl hydrogels was desirable for confirming the strains from the electromechanical signals. The resistance changes of PANaD_0.2_‐NaCl hydrogels with different tensile speeds at a constant strain of 400% were also measured to determine the responsiveness and accuracy of the sensor, as shown in Figure S15c (Supporting Information). PANaD_0.2_‐NaCl hydrogels presented a stable and rapid electromechanical signal response at frequency from 0.15 to 0.53 Hz. PANaD_0.2_‐NaCl hydrogels also showed robust anti‐fatigue performance to ensure signal stability for long‐termed applications. The relative resistance changes of PANaD_0.2_‐NaCl hydrogels remained stable after more than 3600 cycles (2 s per cycle) by repeatedly applying a tensile strain of 250% for 2 h, as shown in Figure 5i. Further cyclic tests were not performed to avoid the potential dehydration. The resistance of PANaD_0.2_‐NaCl hydrogels gradually changed during a tensile deformation from 0 to 3500% strains owing to the superior stretchability of PANaD_0.2_‐NaCl hydrogels (Figure 5j). This result suggested that PANaD_0.2_‐NaCl hydrogels could be applied to sense an extremely large‐scale range of deformations. The gauge factor (GF) of the PANaD_0.2_‐NaCl hydrogels at 25 °C and −20 °C for sensing the strains during the ultra‐large tensile deformations are shown in Figure S16 (Supporting Information). The GF factor gradually increased with the increase in tensile deformations. The GF of PANaD_0.2_‐NaCl hydrogels at −20 °C was lower than that at 25 °C, which should be ascribed to the relatively lower conductivity at lower temperatures.

The resistance changes of PANaD_0.2_‐NaCl hydrogels could be used to reversibly tune the brightness of the light‐emitting diode (LED) in an electric closed‐circuit (Figure 5k; and Video S5, Supporting Information). Meanwhile, the PANaD_0.2_‐NaCl sensors exhibit reliable signal response under −20 °C due to the good anti‐freeze properties (Figure 5c–g,l). Based on the above excellent performance as strain sensors, PANaD_0.2_‐NaCl hydrogels were quite suitable as wearable ionic skins to detect various human activities and health monitoring in real‐life scenarios.

**Figure 6:**
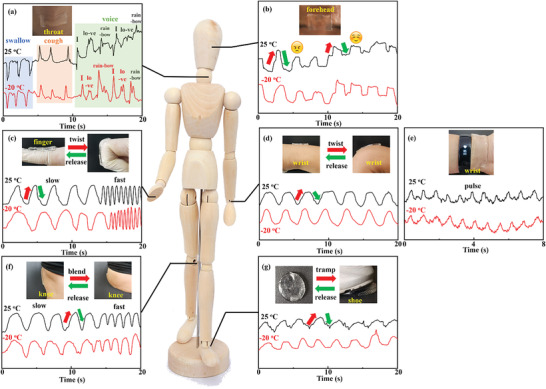
a) Human motion detection of PANaD_0.2_‐NaCl at throat, b) forehead, c) finger, d,e) wrist, f) knee, and g) foot at room temperature and −20 °C.

Skin allergy tests of the hydrogels were performed to test the skin sensitization using the Buehler tests in guinea pigs, according to ISO 10993‐10:2021. Detailed measurement information and the test report are shown in the Supporting Information (Figure S18, Supporting Information). The skin sensitization rate determined from the tests was 0%, which revealed that the PANaD_n_ hydrogels showed no skin allergy for skin sensor applications. As shown in **Figure** **6**, the PANaD_0.2_‐NaCl sensors were directly attached at the forehead, fingers, wrist, knee, and ankle to monitor the motion of humans, such as change of expression, bending of the finger, twist of the wrist, bend of knee, and tramp of foot. Based on the soft feature, the PANaD_0.2_‐NaCl sensor was highly sensitive to the motions, which could be applied to detect slight vibrations, such as swallow, cough and voice at throat, and pulse at wrist (Figure 6a,e). Also, the output signal intensities of the PANaD_0.2_‐NaCl sensors varied obviously by tuning the frequency and amplitude of the motions. The high sensitivity and reliable response of the PANaD_0.2_‐NaCl sensors remained even at a low temperature (−20 °C) due to the good anti‐freeze properties. Based on these results, it could be concluded that the PANaD_0.2_ hydrogel networks had great potential to develop soft, super stretchable, anti‐fracture, and harsh environment adaptive hydrogels for flexible and wearable electronics.

## 3 Conclusion

Herein we proposed a facile and novel approach to prepare unique hydrogels, integrating soft compliance, ultra‐large deformation, robust toughness, and reliable harsh environment adaption. The hydrogel network was simply one‐step constructed via in situ polymerization of aqueous DVB/AANa solutions, wherein DVB was well dispersed by ultrasonic oscillation. The DVB was supposed to provide dynamic hydrophobic associations and homogenous covalent cross‐linking. The dynamic associations of hydrophobic nodes and the full unfolding of polymer chains could work collaboratively to effectively dissipate energy during deformations. The obtained hydrogels showed unique mechanical performance, which were quite soft and deformable (tensile modulus: ≈20 kPa, stretchability: 3700%), but showed excellent anti‐fracture toughness (fracture tensile work: 2.3 MJ m^−3^, toughness: 10.6 kJ m^−2^). The energy dissipation mechanisms were further inspired by the saline or alkaline environments. The mechanical performance of the hydrogel networks was surprisingly promoted rather than weakened under the extreme saline or alkaline environments (stretchability: 5100%, toughness: 16.1 kJ m^−2^ under saturated NaCl environment; and stretchability: 3900%, toughness: 17.1 kJ m^−2^ under 6 mol L^−1^ NaOH environments). The hydrogel network also showed good performance in reversible deformations (3600 th), excellent ion conductivity (20.85 mS cm^−1^), desirable sensing strain and human motions, and good freezing resistance under the extremely harsh environments. The hydrogel network here showed unique outstanding features including soft, super deformable, anti‐fracture and environment adaptable, which highlighted that the present hydrophobic homogenous cross‐linking strategy was a general and scientific principle to develop state‐of‐art hydrogels for diverse applications.

## Conflict of Interest

The authors declare no conflict of interest.

## Supporting information

Supporting Information

Supplemental Video 1

Supplemental Video 2

Supplemental Video 3

Supplemental Video 4

Supplemental Video 5

## Data Availability

The data that support the findings of this study are available from the corresponding author upon reasonable request.
