# Supplementary material for: An Anti‐Fracture and Super Deformable Soft Hydrogel Network Insensitive to Extremely Harsh Environments
Source: Adv Sci (Weinh). 2023 Jun 8;10(23):2302342. doi: 10.1002/advs.202302342 (PMC10427395; doi:10.1002/advs.202302342)
Supplement: Supplementary file 1 — Supporting Information [file ADVS-10-2302342-s006.pdf]

ADVANCED SCIENCE
Open Access

Supporting Information

for *Adv. Sci.*, DOI 10.1002/advs.202302342

An Anti-Fracture and Super Deformable Soft Hydrogel Network Insensitive to Extremely Harsh Environments

*Baibin Yang, Caihong Wang, Ruihan Xiang, Qiang Zhao, Yong Wu and Shuai Tan**

Supporting Information

An anti-fracture and super deformable soft hydrogel network insensitive to extremely harsh environments

*Baibin Yang, Caihong Wang, Ruihan Xiang, Qiang Zhao, Yong Wu, Shuai Tan**

1 Methods

1.1 Chemicals

All the chemical reagents were obtained from TCI and Acros and used as supplied. Deionized water was used for hydrogel preparation.

1.2 Characterization

The Fourier transform infrared (FT-IR) spectra were measured with the use of a Perkin Elmer Spectrum Two Li10014 spectrometer. Before measurements, the hydrogel was freeze-dried under vacuum to a constant weight in a Christ Alpha 1-2LD vacuum freeze dryer. Dynamic light scattering (DLS) analysis was carried out with a Malvern Zetasizer Nano ZS potential analyzer. Atomic force microscopy (AFM) observations were performed on a Cypher VRS. Polarized optical microscope (POM) observations of the stretched hydrogel were performed using a Weitu XPL-30TF POM. Dynamic rheological measurements were performed on a HAAKE MARS60 rheometer at 30 °C using 50 mm flat parallel plates. The samples for dynamic rheological measurements were prepared in the form of a cylinder (ø 50×5 mm). The cylindrical samples were first subjected to a strain sweep test (from 0.1% to 10%) at a constant frequency (ω=1 Hz) to define the linear viscoelastic region. The frequency sweep tests were then performed in the linear regime for all the samples at a strain amplitude of γ=1% over a frequency range of 0.01~100 rad s^{-1}. XRD analyses were carried out by a PANalytical EMPYREAN diffractometer, using Cu-Kα radiation at 40 kV and 40 mA. Relative resistance change tests are carried out by digital multimeter DMM6500. The Differential scanning calorimetry (DSC) measurements were conducted with the use of a TA DSC Q20 modulated instrument with a 1 °C min^{-1} cooling rate from 30 °C to -60 °C under N_2 atmosphere.

The compression and the tension tests were performed using an Instron 5569 electronic universal test machine. The hydrogels were under the as-prepared state. All the tests for the mechanical performance of the hydrogels were performed at room conditions (15~25 ℃, 70~85% relative humidity) without special measures to avoid dehydration of hydrogels. Cylindrical specimens with a size of ø16×10 mm were prepared to perform uniaxial compression tests with a displacement rate of 3 mm min^{-1}. Compressive modulus was calculated from the slope of the linear region of the stress-strain curve during the compression tests. The tension tests and pure shear tests of the samples were performed with a displacement rate of 100 mm min^{-1} without specific description. Dumb-bell-shaped specimens of $PANaD_n$ hydrogels were prepared standardized as ISO 37-2017 (overall length: 50 mm, width of ends: 8.5 mm, length of narrow portion: 16 mm, width of narrow portion: 4 mm, thickness of the sample: 2 mm) for tension tests. Tensile modulus of each stage was calculated from the slope of the linear region of the stress-strain curve during the tension tests. The work (*W*) during the tension process of $PANaD_n$ hydrogels was calculated by integrating the area under the stress-strain curve. In the cyclic tension tests, the crosshead returned to its original position immediately with a displacement rate of 100 mm min^{-1} after the sample was stretched to a 1500% tensile strain with no recovery interval. In the stress relaxation tests, the hydrogels were stretched to a certain strain, then the displacement was held constant. Meanwhile, the force sensor recorded the stress as a function of time. Two identical pieces of hydrogels were prepared with 1.5 mm in thickness and 60 mm in width for the pure shear test. The samples were clamped along the long edges with an initial test length of *H*=10 mm. A notch with a length of 30 mm was introduced into one sample using a razor blade. The fracture energy or toughness (*G*) of $PANaD_n$ hydrogels was calculated from the stress-strain curve of the unnotched sample according to the equation:

$$G=H\int_{1}^{\lambda_c}\sigma d\lambda \qquad \text{(S1)}$$

in which λ_c stands for the critical stretch that the crack propagates for the notched sample. In the tear tests, the samples were prepared with two inextensible backing layers and an initial crack. The two arms of the samples were clamped to perform tear tests and the force sensor recorded the stress at various displacement rates (2-300 mm min^{-1}).

The ionic conductivities of the hydrogels were measured by electrochemical impedance spectroscopy (EIS) with an EG&G Princeton Applied Research P4000+ workstation (Frequency range: 100 kHz~0.01 Hz, applied voltage: 10 mV) using two aluminum foils as electrodes. The

resistance changes of the hydrogels during deformations were recorded by a KEITHLEY DMM6500 6 1/2 digit multimeter. In the section of human motion detections, informed written consent from all participants was obtained prior to the research. During the tests, the hydrogel sensors were only temporarily attached to the skin surface of the participants, rather than implanted in human body.

2. Hydrogel preparation

The PANaD$_n$ hydrogels were one-step prepared *via in-situ* polymerization of sodium acrylate monomer (AANa, 4.5 mol L^{-1}) and DVB (crosslinker, n mol L^{-1}, n=0.1, 0.2, 0.3) aqueous solutions. The aqueous solutions were ultrasonically oscillated for 1 h before polymerization for homogenous dispersion of DVB. DLS measurements revealed that the hydrophobic DVB crosslinker was uniformly dispersed and formed nanodroplets with an average diameter of ~100 nm in AANa solutions **(Figure S2a**). The diameter of DVB nanodroplets was increased with the increase of DVB content at n < 0.3. However, the diameter was almost constant by further adding DVB into solutions, which suggested that the further added DVB was separated from solutions. The maximum content of DVB introduced by ultrasonic oscillation was 0.3 mol L^{-1}. The solution was injected into PTFE molds with specific shapes, and the polymerization was initiated by ammonium persulphate at 60 °C. After 4h, transparent hydrogels PANaD$_n$ with about 70 wt % water were obtained. The water contents of the hydrogels were determined by a gravimetric method. The as-prepared hydrogels were stored at 80 ^{o}C under vacuum, and weighed at appropriate intervals until the weight was constant. The water content was determined by the weight loss divided by the initial weight. The water contents determined by gravimetric methods for PANaD$_{0.1}$, PANaD$_{0.2}$, and PANaD$_{0.3}$ hydrogels were 68.9%, 68.5%, and 67.9, respectively, which were quite close to the calculated water contents (69.4% for PANaD$_{0.1}$, 69.0% for PANaD$_{0.2}$, 68.4% for PANaD$_{0.3}$ hydrogels).

DLS measurements of the as-prepared hydrogels are shown in **Figure S2b**. The PANaD$_{0.2}$ hydrogels were freeze dried to constant weight, then the freeze-dried hydrogel networks were immersed into saturated NaCl solutions or 6 mol L^{-1} NaOH solutions at room temperature until the hydrogel networks recovered to the original volume. The hydrogel swelling NaCl solutions and 6 mol L^{-1} NaOH solutions were labeled as PANaD$_{0.2}$-NaCl and PANaD$_{0.2}$-NaOH, respectively. DLS measurements of the ANaD$_{0.2}$-NaCl and PANaD$_{0.2}$-NaOH hydrogels are shown in **Figure S2c and 2d**.

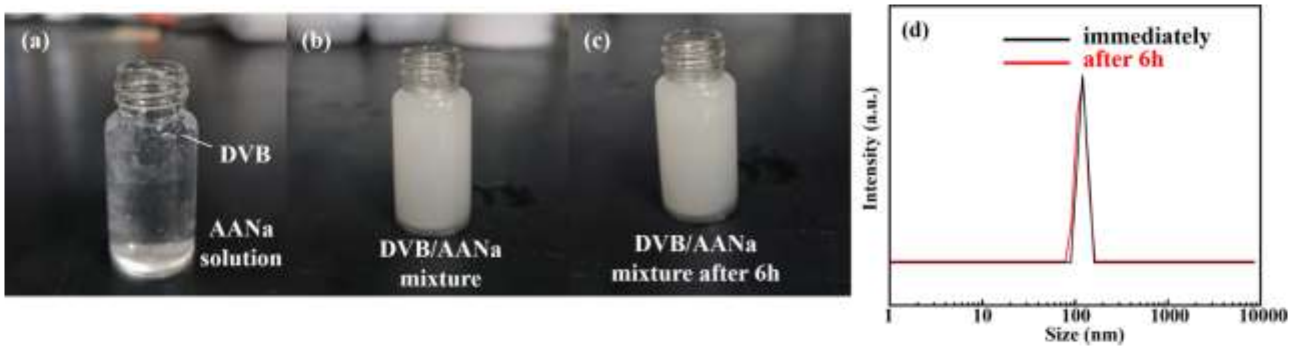

Figure S1 (a) Image of DVB/AANa solution before ultrasonic oscillation, (b) Image of DVB/AANa mixture after ultrasonic oscillation for 1h, (c) Image of DVB/AANa mixture after removing ultrasonic oscillation for 6h, (d) The size distributions from DLS measurements of DVB/AANa mixture at immediately removing ultrasonic oscillation and removing ultrasonic oscillation after 6h.

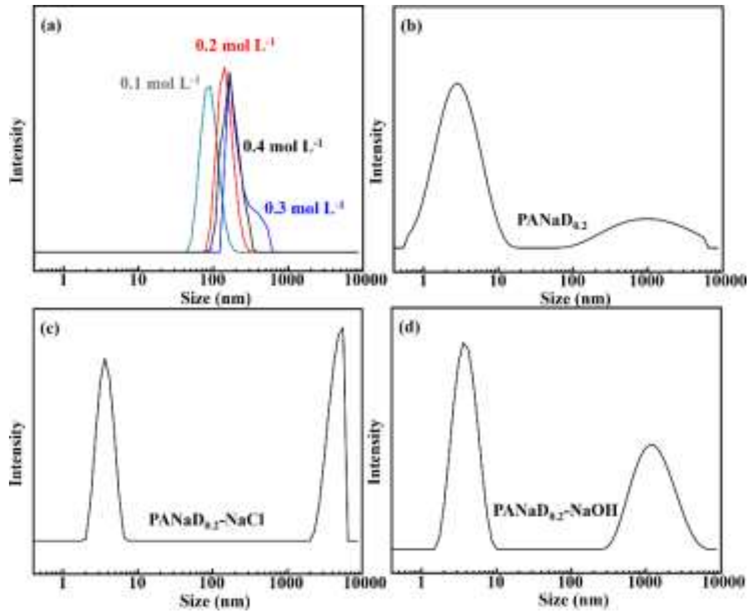

Figure S2. Size distributions of (a) AANa/DVB solutions, (b) PANaD$_{0.2}$ hydrogels, (c) PANaD$_{0.2}$-NaCl hydrogels, and (d) PANaD$_{0.2}$-NaOH hydrogels from DLS measurements

3. AFM image of the PANaD$_{0.2}$ hydrogels

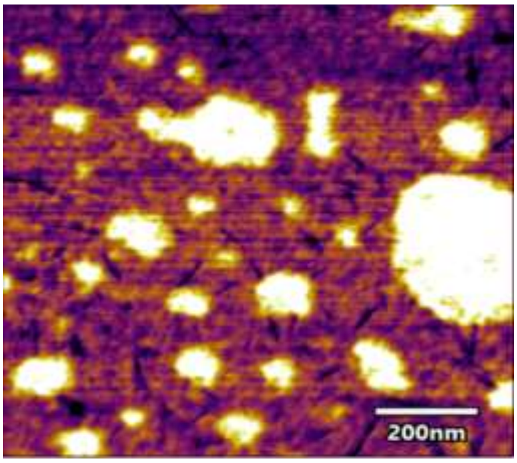

Figure S3. AFM phase diagram of the PANaD$_{0.2}$ Hydrogels.

4. FT-IR spectrum of the PANaD$_{0.2}$ hydrogel network

The FT-IR spectrum of freeze-dried PANaD$_{0.2}$ hydrogel is shown in Figure S4. The absorption peaks at 3320 cm^{-1} and 1550 cm^{-1} were assigned to the vibration of -OH and COO- groups, respectively.[1] The C=C stretching at 1640 cm^{-1} was not observed in the FT-IR spectrum of freeze-dried PANaD$_{0.2}$ hydrogel, which confirmed the successful polymerization of the hydrogels [2].

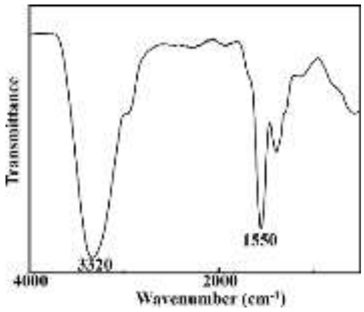

Figure S4. The FT-IR spectra of the PANaD$_{0.2}$ hydrogels.

5. Adhesion performance of the $PANaD_{0.2}$ hydrogels

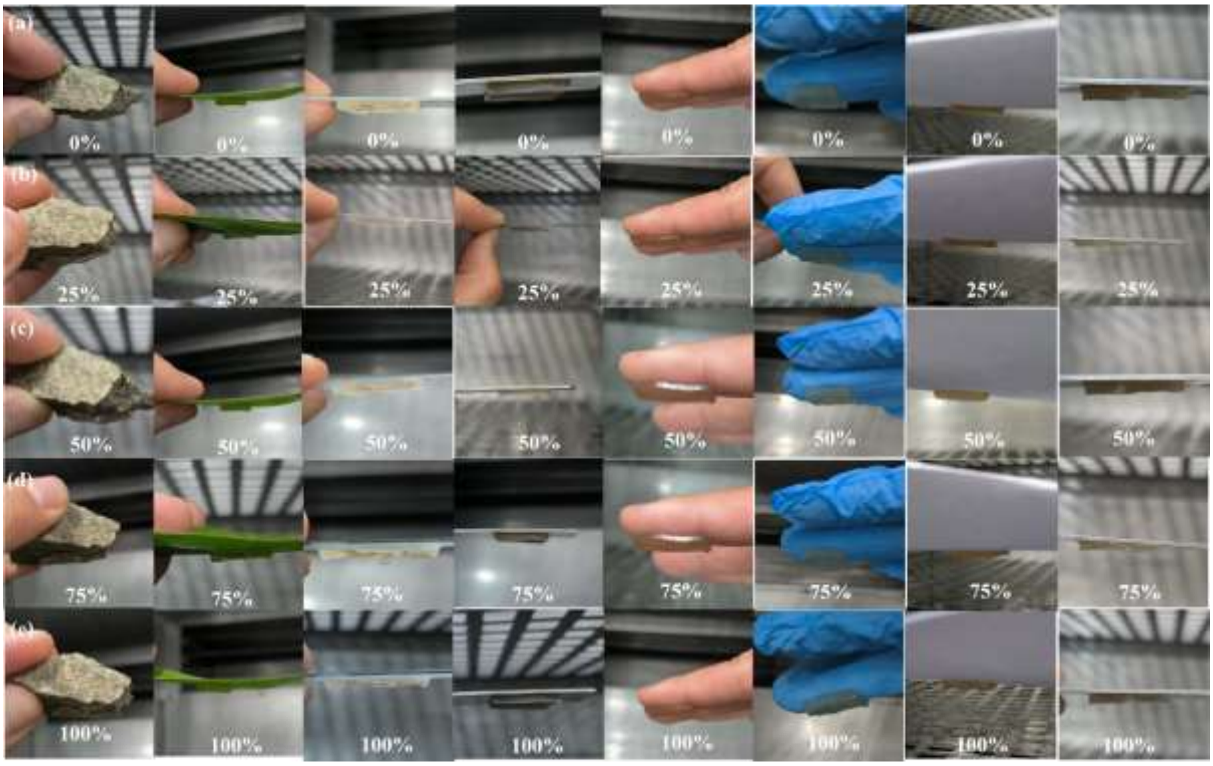

Figure S5. Adhesion performances of the $PANaD_{0.2}$ hydrogels on rock, leaf, glass, steel, skin, latex, paper, and Zinc surfaces (from left to right) under (a) 0%, (b) 25%, (c) 50%, (d) 75%, and (e) 100% humidity.

6. Viscoelastic properties of the $PANaD_n$ hydrogels

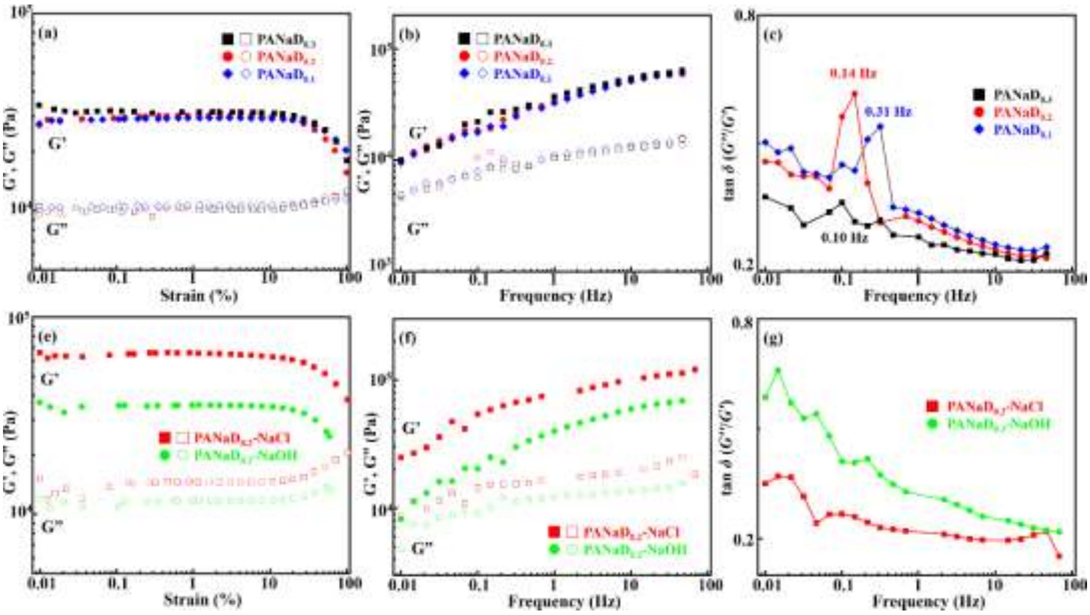

Figure S6. (a) Strain dependence of $PANaD_n$ hydrogels, (b) Frequency dependence of $PANaD_n$

hydrogels, (c) Loss factors tan δ of $PANaD_n$ hydrogels, (d) Strain dependence of $PANaD_{0.2}$-NaCl and $PANaD_{0.2}$-NaOH hydrogels, (e) Frequency dependence of $PANaD_{0.2}$-NaCl and $PANaD_{0.2}$-NaOH hydrogels, and (f) Loss factors tan δ of $PANaD_{0.2}$-NaCl and $PANaD_{0.2}$-NaOH hydrogels.

7. Tear tests of the hydrogels

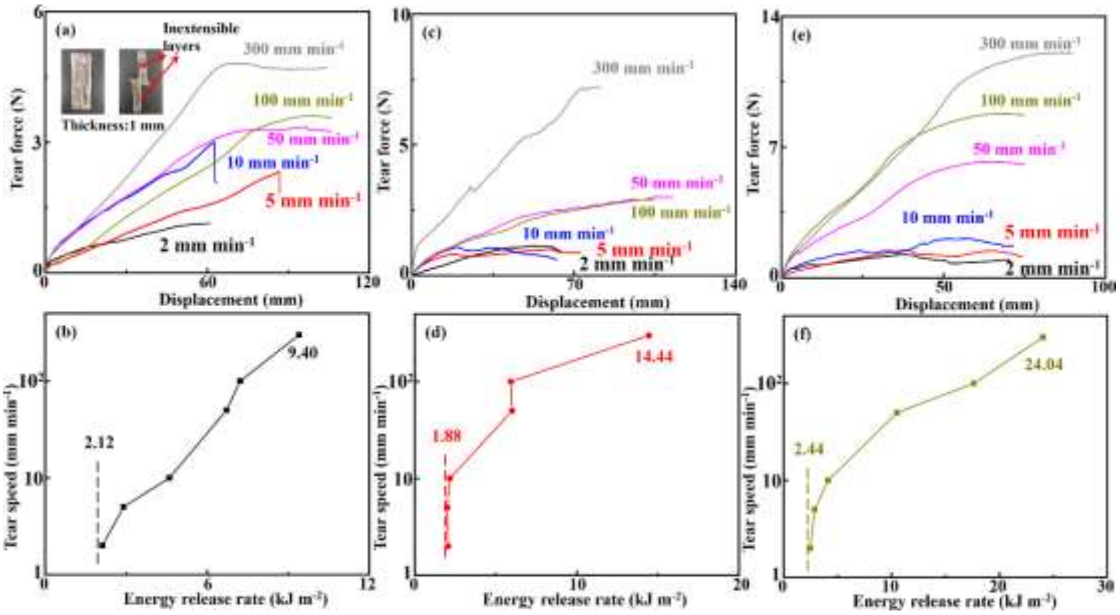

Figure S7. Tear force-displacement curves at various tear speeds for (a) $PANaD_{0.2}$ hydrogels, (c) $PANaD_{0.2}$-NaCl hydrogels, and (e) $PANaD_{0.2}$-NaOH hydrogels; The energy release rates determined from tear tests at various tear speeds for (b) $PANaD_{0.2}$ hydrogels, (d) $PANaD_{0.2}$-NaCl hydrogels, and (f) $PANaD_{0.2}$-NaOH hydrogels

8. POM observations of $PANaD_{0.2}$-NaCl, $PANaD_{0.2}$-NaOH, and reference pure poly (sodium acrylate) hydrogels

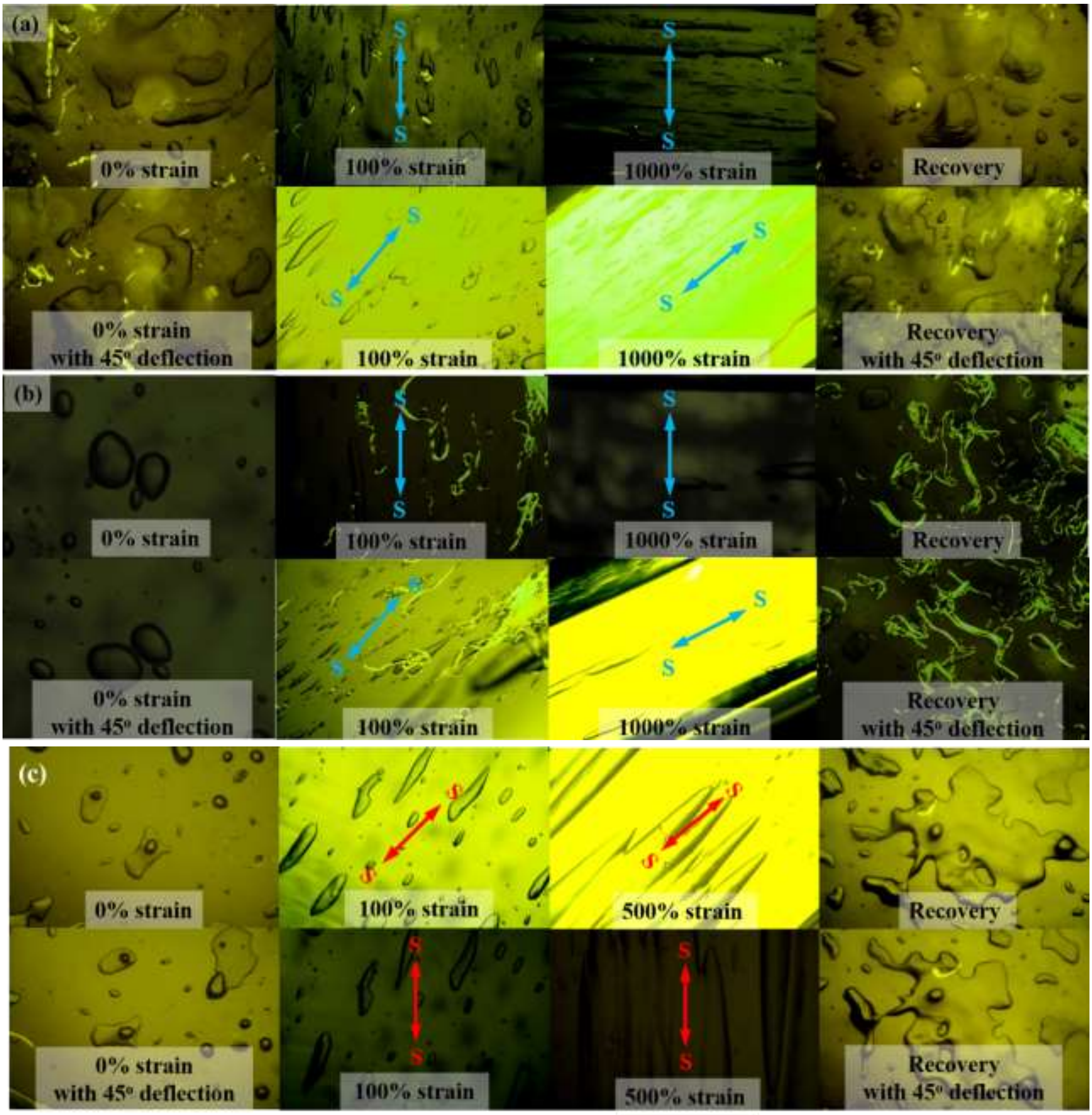

Figure S8. Polarized microscopic image of the (a) $PANaD_{0.2}$-NaCl, (b) $PANaD_{0.2}$-NaOH, and (c) pure poly (sodium acrylate) hydrogels under various states (up) and with 45° deflection (down), S: stretching.

9. Water retention of PANaD$_n$ hydrogels and swelling behavior of freeze-dried PANaD$_n$ hydrogel network

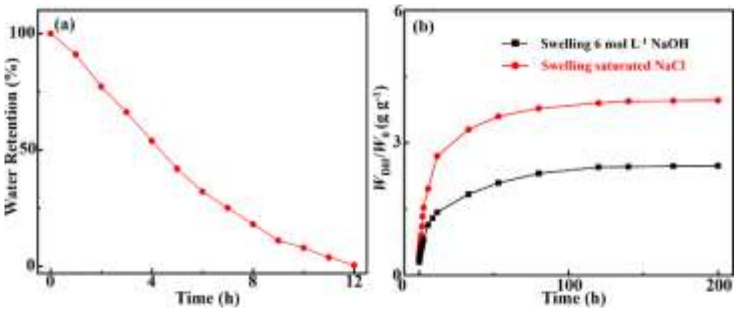

Figure S9. (a) Water retention of PANaD$_n$ hydrogels at 80 °C, (b)Swelling behavior of freeze-dried PANaD$_n$ hydrogel network in saturated NaCl solution and 6 mol L^{-1} NaOH solution. (W_0: initial weight of PANaD$_n$ hydrogel, W_{DH}: weight of PANaD$_n$ network after swelling the solutions for a specific time)

10. Cyclic tensile deformation of the PANaD$_{0.2}$-NaOH hydrogels

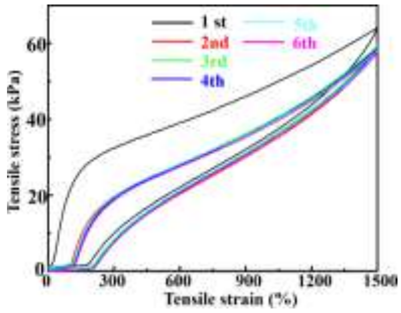

Figure S10. Cyclic tensile stress-strain curves of the PANaD$_{0.2}$-NaOH hydrogels at a 1500% strain with no recovery time

11. Long-termed stability of PANaD$_{0.2}$-NaCl and PANaD$_{0.2}$-NaOH hydrogels

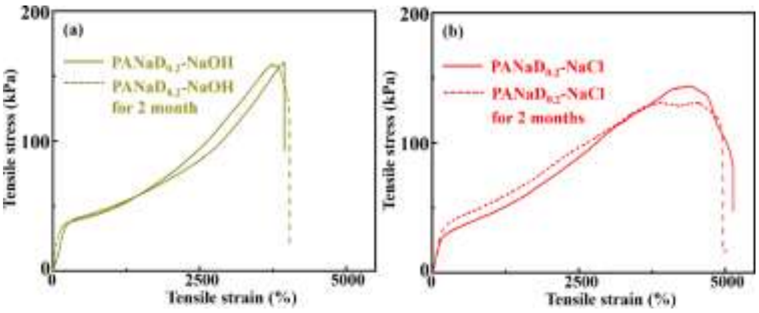

Figure S11. (a) Tensile stress-strain curves of as-prepared PANaD$_{0.2}$-NaOH hydrogels and PANaD$_{0.2}$-NaOH hydrogels after being stored for 2 months, (b) Tensile stress-strain curves of as-prepared PANaD$_{0.2}$-NaCl hydrogels and PANaD$_{0.2}$-NaCl hydrogels after being stored for 2 months

12. XRD measurements of PANaD$_{0.2}$-NaCl and PANaD$_{0.2}$-NaOH hydrogel networks

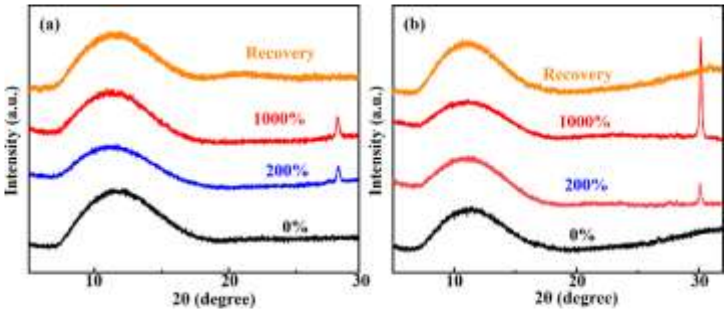

Figure S12. XRD patterns of (a) PANaD$_{0.2}$-NaCl and (b) PANaD$_{0.2}$-NaOH freeze-dried at various states

13. Young's modulus of PANaD$_{0.2}$-NaCl and PANaD$_{0.2}$-NaOH hydrogels at different tensile speeds

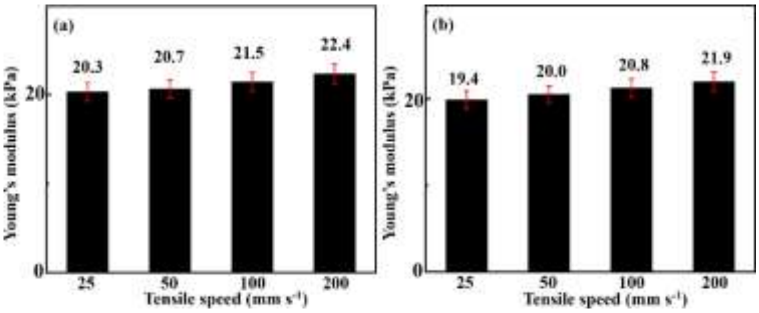

Figure S13. The Young's modulus of PANaD$_{0.2}$-NaCl and PANaD$_{0.2}$-NaOH hydrogels at different tensile speeds.

14.Nyquist plots of PANaD$_{0.2}$-NaCl hydrogels

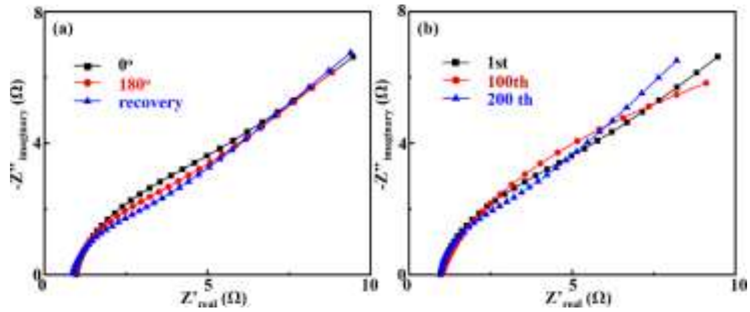

Figure S14. (a) Nyquist plots of PANaD$_{0.2}$-NaCl hydrogels being bended at different degrees, (b) Nyquist plots of PANaD$_{0.2}$-NaCl hydrogels after being bended at 90° for different cycles.

15. Sensing erformance of PANaD$_{0.2}$-NaCl hydrogels

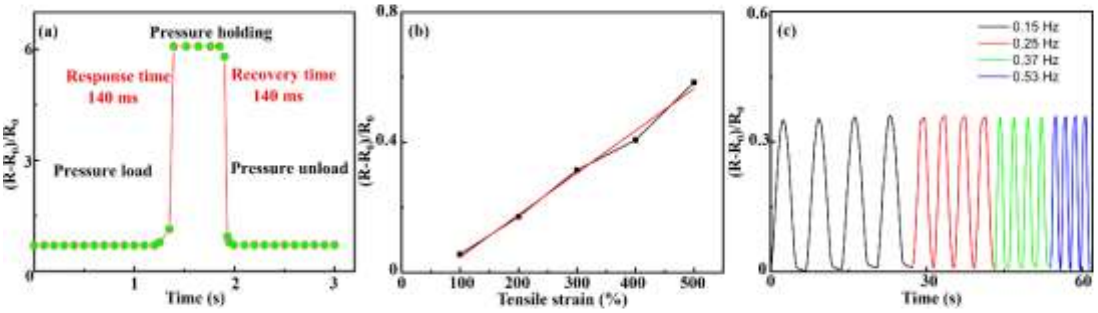

Figure S15. (a) Response and recovery time of sensor based on the PANaD$_{0.2}$-NaCl hydrogels, (b) resistance change as a function of tensile strains, (c) resistance change at different frequencies.

16. GF factors of PANaD$_{0.2}$-NaCl hydrogels

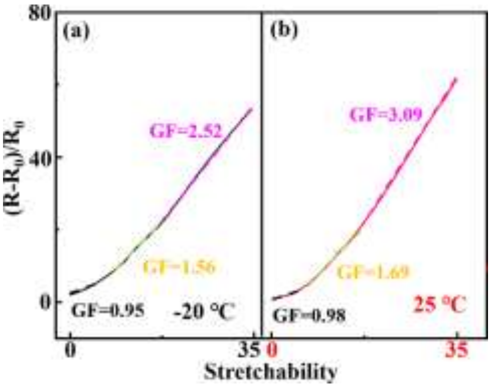

Figure S16. GF factors of the PANaD$_{0.2}$-NaCl hydrogels under a large tensile deformation: (a) -20 ℃, and (b) 25 ℃

17. DSC measurements of the hydrogels

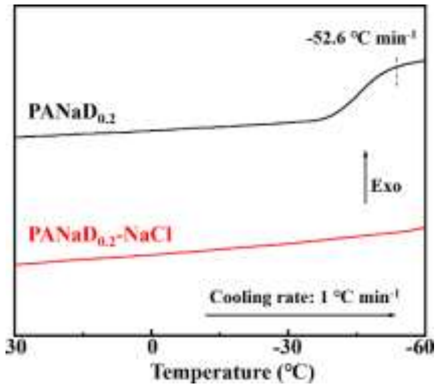

Figure S17. DSC traces of $PANaD_n$ and $PANaD_n$-NaCl hydrogels during cooling

18. Skin allergy tests of the hydrogels

Skin allergy tests were performed to test the skin sensitization of the hydrogels using the Buehler test in guinea pigs, according to ISO 10993-10:2021. The test article and control article were cut into suitable size. The test samples were patched to ten guinea pigs. Five control animals were treated accordingly but with the negative control. The topical challenge with the test article excited no skin reaction in the test and in the control animals. The skin sensitization rate determined from the tests was 0%.

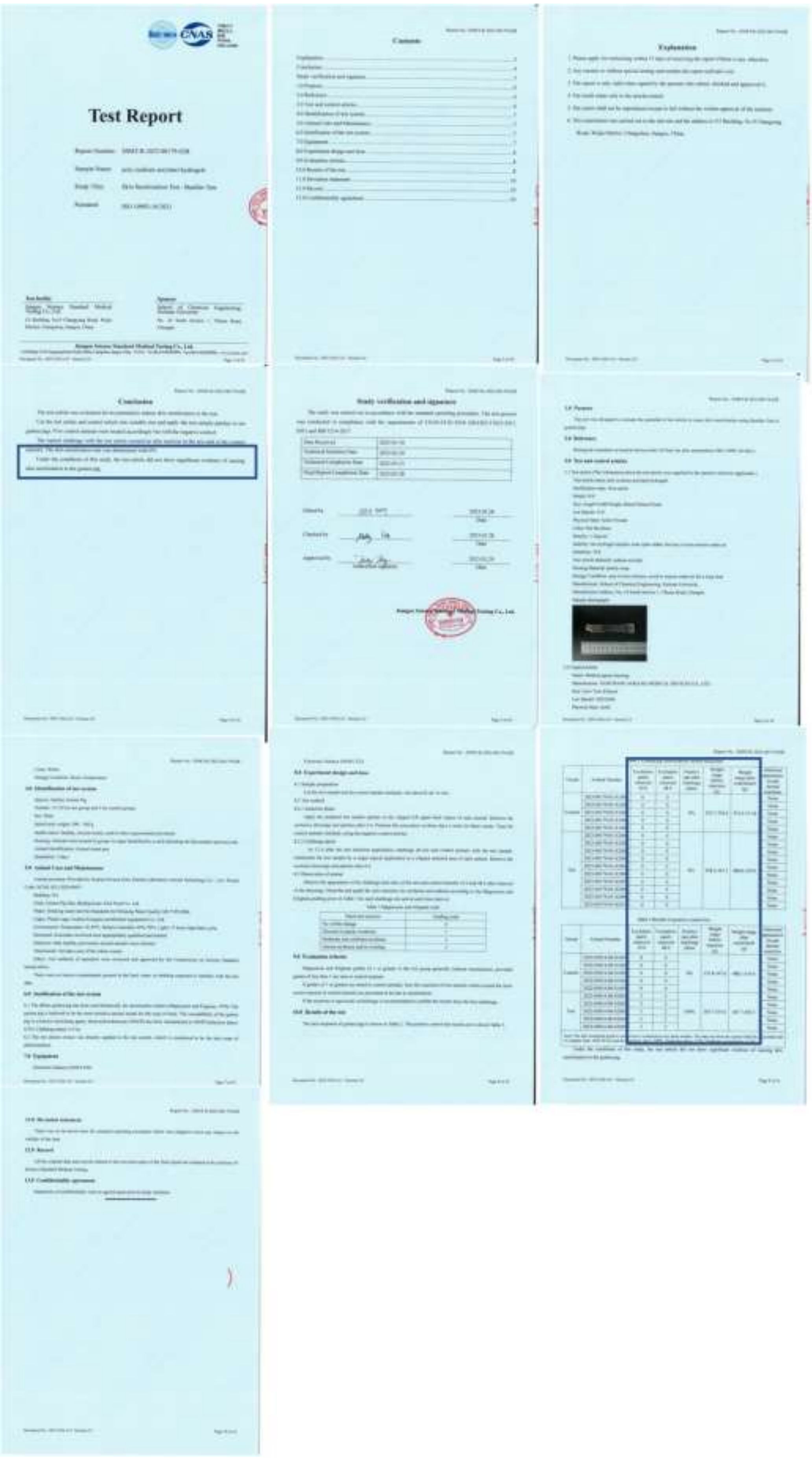

Figure S18. Test report of Skin allergy tests for the hydrogels.

19. Mechanical performance comparison

Table S1. Comparison of PANaD$_{0.2}$ hydrogels with the reported poly (AA) and poly (AANa) hydrogels with extreme mechanical performance in recent five years

Refs	Hydrogels	Young's modulus *E* (kPa)	Stretcha-bility (%)	Fracture Work *W* (MJ m^{-3})	Toughness *G* (kJ m^{-2})	Reversible cyclic deformation	Self-recovery	Adaptation to strong saline or alkaline environments	Anti-freezing
[S3a]	poly (AA) based hydrogels	-[a]	1200	9.8	2.8	-	Yes	Saturated NaCl	-
[S3b]		-	982	0.375	-	-	Yes	-	-
[S3c]		330	574	-	-	-	Yes	2 mol L^{-1} NaCl	-14 °C
[S3d]		3100	2200	18.7	-	-	Yes	-	No
[S3e]		8	1250	0.148	-	100	Yes	-	-14 °C
[S3f]		800	1072	2.484	-	-	Yes	Saturated NaCl	-20 °C
[S3g]		-	2700	-	-	-	-	6 mol L^{-1} KOH	-
[S3h]		2100	420	15.9	-	1000 times	Yes	-	-20 °C
[S3i]		-	2203	4.14	-	-	Yes	-	-
[S3j]		300	800	-	2	-	Yes	-	-
[S3k]		-	981	3.38	-	100 times	Yes	0.15 mol L^{-1} $ZnCl_2$	-
[S3l]		660	1380	30.4	-	-	Yes	-	-
[S3m]		40	589	0.013	-	-	Yes	0.1 mol L^{-1} Fe^{3+}	-
[S3n]		940	1228	25.1	-	-	Yes		-
[S3o]		5630	600	84.7	-	-	Yes	-	-
[S4a]	poly(AANa) hydrogels	-	1700	-	-	-	-	6 mol L^{-1} KOH	-
[S4b]		-	600	-	-	500 times	Yes	-	-
[S4c]		-	1100	-	-	-	-	6 mol L^{-1} KOH	-
[S4d]		-	1000	-	-	-	-	6 mol L^{-1} KOH	-
[S4e]		-	1430	-	-	-	-	6 mol L^{-1} KOH	-
[S4f]		600	325	0.115	-	-	Yes	-	-
**This work**		**20.7**<br>**21.5**[b]<br>**20.8**[c]	**3754**<br>**5100**[b]<br>**3912**[c]	2.3<br>3.7[b]<br>3.2[c]	**10.6**<br>**16.1**[b]<br>**17.8**[c]	**10^4 times at 200% strain**	**Yes**	**Saturated NaCl, 6 mol L^{-1} NaOH**	<-20 °C

a) -: not given, b) under saturated NaCl environment, c) under 6mol L^{-1} NaOH environment

Table S2. Comparison of PANaD$_{0.2}$ hydrogels with the reported hydrogels with extreme mechanical performance

Refs	Energy dissipation mechanism	Stretchab-ility (%)	*E* (kPa)	*G* (kJ m^{-2})	Anti-freezing	Reversible cyclic deformation	Adaptation to strong saline or alkaline environments	Self-recovery
S5a	Freeze-thawed or dry-annealed PVA hydrogels	~260	200	0.1-0.5[a]	No	>30000 th (200% strain)	-[c]	-[c]
S5b		~280	5000	14[a]	-	-	**unstable** in strong alkali	-
S5c		400	10000	10[a]	No	30000 th (200% strain)	-	-
S6a	Double network hydrogels	1200	100	3.8[a]	-	10000 th (200% strain)	**unstable** in strong alkali	-
S6b		700-900	4400-7900	7.7-11.8[b]	-	-	Stable in 2.5 mol L^{-1} NaCl	Yes
S6c		~800	3000-3500	-	-	-	-	Yes
S6d		450	1300	14[a]	-	-	Stable in saturated Na_2SO_4 and Na_3Cit solutions	Yes
S6e		800	2400	8[b]	-	-	**unstable** in salinity	Yes
S6f		500-2500	100-1000	4-16[a]	-	-	-	-[c]
S6g		600	56000	-	-	-	-	Yes
S7a	Polyampholytes	~1400	140	-	-	50000 th (320% strain)	-	Yes

S7b		~700	100-2200	~4[ab]	-	-	**unstable** in salinity	Yes
S8	Elastomer-hydrogel composite	200-400	200	4.5[a]	-	30000 th (170% strain)	**unstable** in strong alkali	-
S9	Supramolecular polymer hydrogel	1400	150	1.1[b]	-	-	**unstable** in strong alkali	Yes
S10	Ion interaction	600	15400	22.1[a]	-	-	**unstable** in strong alkali	Yes
S11	Highly-entangled hydrogel	550	100-200	1.46[a]	-	200000 th	-	-
S12	polyprotein cross-linkers	1100	12	0.9[a]	-	5000 th (500% strain)	-	-
S13	Topoarchitected polymer networks	470	40	4.5[a]	-	5000 th (50% strain)	-	-
S14	Nanocomposition	1500	-	-	-20 ^{o}C; anti-drying	-	-	Yes
S15	Organohydrogels	2500	-	-	-55 ^{o}C; anti-drying	-	Stable in 2M NaCl	
This work	hydrophobic homogenous crosslinking	**3754**<br>**5100**[d]<br>**3912**[e]	**20.7**<br>**21.5**[d]<br>**20.8**[e]	**10.6**[a]<br>**16.1**[ad]<br>**17.8**[ae]	**-20 ^{o}C**	**10000 th (200% strain)**	**Stable in Saturated NaCl, 6 mol L^{-1} NaOH**	**Yes**

a) from pure shear tests b) from tear test, c) -: not given, d) under saturated NaCl environment, e) under 6mol L^{-1} NaOH environment

References

[S1] Y. Zhang, Y. Chen, M. Alfred, F. Huang, S. Liao, D. Chen, D. Li, Q. Wei, *Compos. Part B-Eng* **2021**, *224*, 109228.

[S2] I. C. Li, Y.H. Chen, Y.-C. Chen, *J. Water Process. Eng.* **2022**, *49*, 102999.

[S3] a) X.Wang, F. Song, D. Qian, Y. He, W. Nie, X. Wang, Y. Wang, *Chem. Eng. J.* **2018**, *349*, 588; b) Y. Jiao, Y. Lu, K. Lu, Y. Yue, X. Xu, H. Xiao, J. Li, J. Han, *J. Colloid Interface Sci.* **2021**, *597*, 171; c) S. Li, H. Pan, Y. Wang, J. Sun, *J. Mater. Chem. A* **2020**, *8*, 3667; d) M. Wang, Y. Chen, Y. Gao, C. Hu, J. Hu, L. Tan, Z. Yang, *ACS Appl. Mater. Interfaces* **2018**, *10*, 26610; e) Z. He, W. Yuan, *ACS Appl. Mater. Interfaces* **2021**, *13*, 1474; f) G. Chen, J. Huang, J. Gu, S. Peng, X. Xiang, K. Chen, X. Yang, L. Guan, X. Jiang, L. Hou, *J. Mater. Chem. A* **2020**, *8,* 6776; g) K. Tang, C. Yuan, Y. Xiong, H. Hu, M. Wu, *Appl. Catal. B Environ.* **2020**, *260,* 118209; h) H. Liu, X. Wang, Y. Cao, Y. Yang, Y. Yang, Y. Gao, Z. Ma, J. Wang, W. Wang, D. Wu, *ACS Appl. Mater. Interfaces* **2020**, *12*, 25334; i) X. Jiang, N. Xiang, J. Wang, Y. Zhao, L. Hou, *Carbohydr Polym.* **2017**, *173*, 701; j) M. Milovanovic, N. Isselbaecher, V. Brandt, J. C. Tiller, *Chem. Mater* **2021**, *33*, 8312; k) Y. Wu, Y. Zeng, Y. Chen, C. Li, R. Qiu, W. Liu, *Adv. Funct. Mater* **2021**, *31*, 2107212; l) Y. Wang, X. Fang, S. Li, H. Pan, J. Sun, *ACS Appl Mater Interfaces* **2021**, DOI: 10.1021/acsami.1c21002; m) T. Zhang, T. Zuo, D. Hu, C. Chang, *ACS Appl. Mater. Interfaces* **2017**, *9*, 24230; n) X. Li, H. Wang, D. Li, S. Long, G. Zhang, Z. Wu, *ACS Appl. Mater. Interfaces* **2018**, *10*, 31198; o) J. Cao, J. Li, Y. Chen, L. Zhang, J. Zhou, *Adv. Funct. Mater* **2018**, *28*, 1800739.

[4] a) J. Liu, M. Hu, J. Wang, N. Nie, Y. Wang, Y. Wang, J. Zhang, Y. Huang, *Nano Energy* **2019**, *58*, 338; b) S. Zhang, Y. Li, H. Zhang, G. Wang, H. Wei, X. Zhang, N. Ma, *ACS Mater. Lett.* **2021**, *3*, 807; c) L. Ma, S. Chen, D. Wang, Q. Yang, F. Mo, G. Liang, N. Li, H. Zhang, J. A. Zapien, C. Zhi, *Adv. Energy Mater.* **2019**, *9*, 1803046; d) Y. Huang, J. Liu, J. Wang, M. Hu, F. Mo, G. Liang, C. Zhi, *Angew. Chem. Int. Ed. Engl.* **2018**, *57*, 9810; e) D. Wang, Z. Li, L. Yang, J. Zhang, Y. Wei, Q. Feng, Q. Wei, *Chem. Eng. J.* **2023**, *454*, 140090; f) J. Lai, H. Zhou, M. Wang, Y. Chen, Z. Jin, S. Li, J. Yang, X. Jin, H. Liu, W. Zhao, *J. Mater. Chem. C* **2018**, *6*, 13316.

[S5] a) S. Lina, J. Liu, X. Liu, X. Zhao, *Proc. Natl. Acad. Sci. U. S. A.* **2019**, *116*, 10244; b) J. Li, Z. Suo, J. Vlassak, *J. Mater. Chem. B*, **2014**, *2*, 6708; c) S. Lin, X. Liu, H. Yuk, H. Loh, G. A. Parada, C. Settens, J. Song, A. Masic, G. H. Mckinley, X. Zhao, *Sci. Adv.* **2019**, *5*, eaau8528.

[S6] a) W. Zhang, X. Liu, J. Wang, J. Tang, J. Hu, T.Lu, Z. Suo, *Eng. Fract. Mech.* **2018**, *18*7, 74; b) F. Luo, T. Sun, T. Nakajima, T. Kurokawa, Y. Zhao, K. Sato, A. Ihsan, X. Li, H. Guo, J. Gong, *Adv. Mater.* **2015**, *27*, 2722; c) P. Lin, S. Ma, X. Wang, F. Zhou, *Adv. Mater.* **2015**, *27*, 2054; d) Y. Yang, X. Wang, F. Yang, L. Wang, D. Wu, *Adv. Mater.* **2018**, *30*, 1707071; e) H. Chen, Y. Liu, B. Ren, Y. Zhang, J. Ma, L. Xu, Q. Chen, J. Zheng, *Adv. Funct. Mater.* **2017**, *27*, 1703086; f) J. Li, W. Illeperuma, Z. Suo, J. Vlassak, *ACS Macro Lett.* **2014**, *3*, 520; g) J. Cao, J. Li, Y. Chen, L. Zhang, J. Zhou, *Adv. Funct. Mater.* **2018**, *28*, 1800739.

[S7] a) X. Li, K. Cui, T. Sun, L. Meng, C. Yu, Li. Li, C. Creton, T. Kurokawa, J. Gong, *Proc. Natl. Acad. Sci. U. S. A.* **2020**, *117*, 7606; b) T. Sun, T. Kurokawa, S. Kuroda, A. Ihsan, T. Akasaki, K. Sato, M. Haque, T. Nakajima, J. Gong, *Nat. Mater.* **2013**, *12*, 932.

[S8] C. Xiang, Z. Wang, C. Yang, X. Yao, Y. Wang, Z. Suo, *Material Today*, **2020**, *34*, 7.

[S9] X. Dai, Y. Zhang, L. Gao, T. Bai, W. Wang, Y. Cui, W. Liu, *Adv. Mater.* **2015**, *27*, 3566.

[S10] C. Jiao, J. Zhang, T. Liu, X. Peng, H. Wang, *ACS Appl. Mater. Interfaces* **2020**, *12*, 44205.

[S11] J. Kim, G. Zhang, M. Shi, Z. Suo, *Science* **2021**, *374*, 212.

[S12] H. Lei, L. Dong, L. Li, J. Zhang, H. Chen, J. Wu, Y. Zhang, Q. Fan, B. Xue, M. Qin, B. Chen, Y. Cao, *Nat. Commun.* **2020**, *11*, 4032.

[S13] X. Liu, J. Wu, K. Qiao, G. Liu, Z. Wang, T. Lu, Z. Suo, J. Hu, *Nat. Commun.* **2022**, *13*, 1622.

[S14] S. Li, Z. Yu, B, Guo, K, Guo, Y. Li, L. Gong, L. Zhao, J. Bae, L. Tang, *Nano Energy* **2021**, *90*, 106502.

[S15] Y. Wu, J. Qu, X. Zhang, K. AO, Z. Zhou, Z. Zheng, Y. Mu, X. Wu, Y. Luo, S. Feng, *ACS Nano* **2021**, *15*, 13427.
